# A distinct complex of PRP19-related and trypanosomatid-specific proteins is required for pre-mRNA splicing in trypanosomes

**DOI:** 10.1093/nar/gkab1152

**Published:** 2021-12-01

**Authors:** Ankita Srivastava, Daniela L Ambrósio, Monika Tasak, Ujwala Gosavi, Arthur Günzl

**Affiliations:** Department of Genetics and Genome Sciences, University of Connecticut Health Center, 400 Farmington Avenue, Farmington, CT 06030-6403, USA; Department of Genetics and Genome Sciences, University of Connecticut Health Center, 400 Farmington Avenue, Farmington, CT 06030-6403, USA; Departamento de Ciências da Biointeração, Universidade Federal da Bahia, Canela, Salvador, 40231-300, Brazil; Department of Genetics and Genome Sciences, University of Connecticut Health Center, 400 Farmington Avenue, Farmington, CT 06030-6403, USA; Department of Genetics and Genome Sciences, University of Connecticut Health Center, 400 Farmington Avenue, Farmington, CT 06030-6403, USA; Department of Genetics and Genome Sciences, University of Connecticut Health Center, 400 Farmington Avenue, Farmington, CT 06030-6403, USA

## Abstract

The pre-mRNA splicing factor PRP19 is recruited into the spliceosome after forming the PRP19/CDC5L complex in humans and the Nineteen complex in yeast. Additionally, ‘PRP19-related’ proteins enter the spliceosome individually or in pre-assemblies that differ in these systems. The protistan family Trypanosomatidae, which harbors parasites such as *Trypanosoma brucei*, diverged early during evolution from opisthokonts. While introns are rare in these organisms, spliced leader *trans* splicing is an obligatory step in mRNA maturation. So far, ∼70 proteins have been identified as homologs of human and yeast splicing factors. Moreover, few proteins of unknown function have recurrently co-purified with splicing proteins. Here we silenced the gene of one of these proteins, termed PRC5, and found it to be essential for cell viability and pre-mRNA splicing. Purification of PRC5 combined with sucrose gradient sedimentation revealed a complex of PRC5 with a second trypanosomatid-specific protein, PRC3, and PRP19-related proteins SYF1, SYF3 and ISY1, which we named PRP19-related complex (PRC). Importantly, PRC and the previously described PRP19 complex are distinct from each other because PRC, unlike PRP19, co-precipitates U4 snRNA, which indicates that PRC enters the spliceosome prior to PRP19 and uncovers a unique pre-organization of these proteins in trypanosomes.

## INTRODUCTION

*Trypanosoma brucei* and related organisms of the phylogenetic family Trypanosomatidae have streamlined genomes in which protein-coding genes are arranged in dense directional arrays. These gene arrays are transcribed polycistronically, and individual mRNAs are processed from pre-mRNA by spliced leader (SL) *trans* splicing and polyadenylation. In *trans* splicing, the SL, also referred to as the mini-exon, is transferred from the 5’ end of the small nuclear SL RNA to the 5’ end of each mRNA (1,2). Mechanistically, SL *trans* splicing is achieved by the same two consecutive transesterifications as *cis* splicing, i.e. the removal of introns (3,4). While *T. brucei* processes every pre-mRNA by *trans* splicing, only the pre-mRNA of the poly(A) polymerase PAP1 (5) and the DEAD box helicase DBP2B are also *cis*-spliced, harboring two exons separated by a single intronic sequence (6,7).

Pre-mRNA splicing is carried out by the spliceosome, a large and dynamic complex consisting of the U-rich (U) small nuclear (sn)RNAs U1, U2, U4, U5 and U6, and about 85 core spliceosomal proteins that are conserved between humans and the budding yeast *Saccharomyces cerevisiae*. In addition, the spliceosome comprises system-specific components, which, in humans, comprise ∼80 additional proteins (8,9). The U snRNAs directly bind to ∼45 proteins, forming small nuclear ribonucleoprotein complexes or snRNPs. The remaining non-snRNP proteins enter the spliceosome independently or as preformed complexes. The spliceosome assembles step-by-step on pre-mRNA guided by the 5’ splice site, the branch point (BP) which often is adjacent to and upstream of a polypyrimidine tract, and the 3’ splice site. Initially, the spliceosomal E (early) complex is formed in which the 5’ splice site is recognized by the U1 snRNP, the BP by the branch point binding protein SF1 (splicing factor 1), and polypyrimidine tract and 3’ splice site by the U2-auxillary factor (U2AF). Recruitment of the U2 snRNP to BP and displacement of SF1 lead to the A complex, and further recruitment of the U4/U6.U5 tri-snRNP to the precatalytic B complex that harbors all five snRNAs. Activation of the spliceosome, i.e. formation of the B^act^ spliceosome, requires the release of U1 and U4 snRNPs. After the first transesterification, complex C is formed and after the second transesterification, the spliceosome is disassembled and its components recycled for the next splicing event.

Several non-snRNP proteins are essential for these transitions of the spliceosome and for proper splicing to occur. A key factor is the protein PRP19 that assembles into a stable non-snRNP protein complex, and, in addition, is associated with so-called ‘PRP19-related’ proteins that either interact with the PRP19 complex in humans or yeast or were detected together with PRP19 in the human 35S U5 snRNP (10–13). These proteins enter the spliceosome before activation and are typically required for the first transesterification to occur. Interestingly, pre-organization of these proteins is different between the human and yeast systems. In humans, two distinct complexes are formed, namely the PRP19/CDC5L complex, which consists of seven subunits (14), and the pentameric intron-binding complex (IBC) that includes SYF1, ISY1 and the RNA helicase Aquarius (15). Additional PRP19-related proteins such as SYF3, SKIP and PPIL1 seem to be recruited to the human spliceosome independently (8). In contrast, yeast PRP19 is part of the larger Nineteen Complex (NTC) that comprises homologs of some but not all of the human PRP19 and IBC subunits as well as some of the proteins that are independently recruited in the human system (13,16,17).

Around 70 spliceosomal proteins have been identified in *Trypanosoma brucei* so far (1,18,19). According to this set of proteins, trypanosomes have homologs of most human snRNP proteins whereas the repertoire of spliceosomal non-snRNP proteins is limited, likely because intact spliceosomes could not be isolated from trypanosome extracts so far (1). Accordingly, characterization of the non-snRNP PRP19 complex identified new splicing factors in trypanosomes. Tandem affinity purification (TAP) of tagged PRP19 combined with sucrose gradient sedimentation revealed a complex of seven co-sedimenting proteins. Besides PRP19 they included CDC5, SPF27 and PRL1, which form the core of the human PRP19/CDC5L complex and are part of the yeast NTC, as well as homologs of human SKIP, PPIL1 and the B^act^–specific protein PRP17 which are not considered subunits of human and *S. cerevisiae* PRP19 complexes, suggesting trypanosome-specific pre-organization of PRP19-associated proteins (19). Structural and functional studies in human and yeast systems have shown that PRP19 and associated proteins are essential for the first splicing step; they interact predominantly with U6 snRNA, stabilizing its association with the B^act^ spliceosome (10,20–24). Moreover, it has been postulated that these proteins maintain the catalytically active conformation of the spliceosome (8). Accordingly, conditional silencing of trypanosome *SPF27* interfered with the first splicing step (19), and RNA immunoprecipitation of PRP19 or its partners CDC5 and SPF27 resulted in strong signals of U2, U5 and U6 snRNAs whereas U1 and U4 snRNA were hardly detectable (19). These results confirmed that spliceosome activation in trypanosomes requires the discard of U1 and U4 snRNAs as in other systems, and it indicated that the PRP19 complex enters the spliceosome during the activation stage.

Although splicing factors in trypanosomes are divergent in sequence from their human and yeast counterparts, they typically exhibit enough sequence similarity for unambiguous identification. However, a few proteins of unknown function have consistently co-purified with spliceosomal complexes. For example, the proteins of *T. brucei* genes *Tb927.11.2960* [gene accession number at TriTrypDB.org (25)] and *Tb927.2.3400* were consistently co-isolated with the common snRNP protein SmD1 (26) and with PRP19 (19), which suggested that they are splicing proteins. Accordingly, both of these proteins localized to the nucleoplasm in the ongoing TrypTag project (http://tryptag.org) in which all *T. brucei* proteins are tagged and localized (27). Moreover, both genes are restricted to trypanosomatids and are seemingly absent from the genome of the closely related kinetoplastid organism *Bodo saltans*, strongly indicating that they encode proteins specific to this family of parasites. Here we show that conditional silencing of *Tb927.11.2960* was lethal to cultured trypanosomes, causing a pre-mRNA splicing defect. TAP and co-sedimentation of the protein identified a pentameric protein complex that included the PRP19-related proteins SYF1, SYF3 and ISY1, as well as the protein encoded by gene *Tb927.2.3400*. Since the complex did not harbor an RNA helicase, i.e. an Aquarius homolog, it remains unclear whether this complex is equivalent to the human IBC complex. We therefore tentatively named this complex ‘PRP19-related complex’ or PRC and the two trypanosomatid-specific proteins according to their size rank within the complex PRC3 (Tb927.2.3400) and PRC5 (Tb927.11.2960). Although the three conserved subunits of the PRC are part of the yeast NTC, we provide evidence that trypanosome PRC and PRP19 are distinct complexes that enter the spliceosome at different stages rather than being subcomplexes of a trypanosome NTC.

Furthermore, *T. brucei* is a parasite that is transmitted by the tsetse fly and lives freely in the bloodstream of its mammalian host. The main proliferating stages are procyclic trypanosomes in the fly and long slender bloodstream trypanosomes in the mammalian host. Interestingly, our gene silencing data suggest that *PRC3* is indispensable in bloodstream trypanosomes while its efficient knockdown in procyclic trypanosomes resulted only in a moderate growth defect.

## MATERIALS AND METHODS

### DNA

Plasmids PRC5-PTP-NEO and PRC3-PTP-NEO were generated by inserting, respectively, 235 bp (nucleotide positions 90–324 relative to the adenine residue of the initiation codon) and 489 bp (positions 661–1149) of the 3′-terminal coding regions into the pC-PTP-NEO tagging vector (28), using the ApaI and NotI restriction sites. Two silent mutations, A210G and G213T, were introduced into the *PRC5* sequence to generate a StuI linearization site within the coding region. Plasmids T7-PRC5-stl and T7-PRC3-stl were generated for conditional expression of hairpin RNAs that target *PRC5* and *PRC3* mRNAs via the RNAi pathway, respectively. For pT7-PRC5-stl 298 bp of PRC5 coding region (positions 104–402) and for pT7-PRC3-stl 520 bp of PRC3 coding region (positions 20–540) were inserted in a published stem-loop arrangement (29) into the pT7-stl vector (30) using the MluI and HindIII restriction sites.

### Cells

Procyclic *Trypanosoma brucei* strain 427 and genetically modified cell lines derived from this strain, such as the 29–13 cell line, were grown in SDM-79 medium at 28°C and bloodstream trypanosomes of the ‘single marker’ cell line (smBT) and its derivative lines were grown in HMI medium at 37°C as detailed previously (31,32). Genetically modified, clonal cell lines were obtained by transfection of linearized plasmids or DNA amplification products by electroporation, and subsequent cloning by limiting dilution as previously described (33,34). The TbPRC5-PTPee procyclic line was generated by integrating StuI-linearized pPRC5-PTP-NEO into one *PRC5* allele. In a second step, the remaining wild-type *PRC5* allele was deleted with a PCR product that contained the hygromycin phosphotransferase (HPT) coding region surrounded by 100 bp of *PRC5* 5’ and 3’ gene flanks. Procyclic lines that exclusively express SmD1-PTP and PRP19-PTP were described previously (19,26). TbPRC3-PTP cells were obtained by transfection of SmaI-linearized pPRC3-PTP-NEO. For conditional gene silencing experiments, EcoRV-linearized pT7-PCR5-stl and pT7-PCR3-stl, both containing a phleomycin resistance gene, were transfected into 29–13 and smBT cells, targeting the plasmids for integration to the transcriptionally silent spacer of rRNA gene units. Dependent on the introduced resistance genes, procyclic trypanosomes were grown with either G418, hygromycin or phleomycin at final concentrations of 40, 40 and 2.5 μg/ml, respectively, whereas the phleomycin concentration in bloodstream trypanosome cultures was 1 μg/ml. For each stable transfection, correct integration of transfected DNA was determined by PCR of genomic DNA with at least one oligonucleotide hybridizing outside the cloned or amplified sequence. Gene silencing of smBT and 29–13 derivative cell lines was induced by adding doxycycline to the medium at a final concentration of 2 μg/ml, and culture growth curves in the absence and presence of doxycycline were generated by counting and diluting cells daily to 2 × 10^6^ cells/ml for procyclic and to 2 × 10^5^ cells/ml for bloodstream trypanosomes.

### RNA analysis

For RNA analyses, total RNA of 10^8^ procyclic or 5 × 10^7^ bloodstream trypanosomes was prepared using the Trizol reagent (Thermo Fisher Scientific) according to the manufacturer’s protocol. To detect rRNA, 2 μg of total RNA was electrophoretically separated in reliant precast 1.25% SeaKem Gold agarose RNA gels (Lonza) and stained with ethidium bromide. Primer extension reactions were carried out with 1 μg of total RNA, DNA oligonucleotides that either carried a ^32^P-radiolabeled phosphate or a biotin group at their 5’ end (Supplementary Table S1 lists all oligonucleotides used in RNA analysis), and SuperScript II reverse transcriptase (Thermo Fisher Scientific) according to the manufacturer’s protocol. Radiolabeled extension products were separated on 50% urea–8% polyacrylamide gels and visualized by autoradiography, whereas biotinylated extension products were separated on 50% urea-6% polyacrylamide gels, electroblotted onto positively charged nylon membrane (Roche) and developed with the Chemiluminescent Nucleic Acid Detection Module Kit (Thermo Fisher Scientific) according to the manufacturer’s specifications. Primer extension signals were quantified by densitometry using the ImageJ software package (35). For reverse transcription (RT)-PCR analyses, 1 μg of total RNA was reverse transcribed with SuperScript IV and either random hexamers (Roche) or Oligo(dT)_18_. For semiquantitative PCR, the number of cycles of the linear amplification range was determined empirically for each oligonucleotide pair. Quantitative (q)PCR reactions were carried out in triplicate on a CFX96 cycler (Biorad) using SsoFast EvaGreen Supermix (Biorad). Suitability of each oligonucleotide pair for qPCR was ensured by agarose gel electrophoresis and a melting curve analysis. In addition, for each qPCR reaction, a standard curve of serially diluted cDNA was co-analyzed and the results only taken into account when the coefficient of determination, *R*^2^, was in the range between 0.98 and 1.0. To detect SL *trans* and *cis* splicing defects, i.e. distinct products of mature mRNA and unspliced pre-mRNA, random hexamer-derived cDNA was amplified standardly by 35 PCR cycles with three or two primers as detailed under Results.

### Protein analysis

Based on the composite PTP tag that consists of the Protein C epitope (ProtC), a tobacco etch virus (TEV) protease site, and tandem protein A domains (ProtA), PRC5-PTP tandem affinity purification (TAP) was carried out exactly according to the standard protocol (28,36). Consecutively, it comprised IgG affinity purification, release of the tagged protein by TEV protease cleavage, in which PRC5-PTP was reduced to PRC5-P, calcium ion-dependent anti-ProtC immunoaffinity purification and final elution through calcium chelation. The efficiency of each purification step was monitored by immunoblotting in which tagged PRC5 was detected with the monoclonal anti-ProtC antibody HPC-4 (Roche) at 1:2000 dilution and a secondary anti-mouse IgG antibody (ThermoFisher) at 1:5000 dilution. Copurified proteins were visualized by electrophoretic separation in a 10–20% gradient SDS polyacrylamide gel (BioRad) and staining with Coomassie Blue (Gelcode Coomassie stain, Thermo Fisher Scientific) or SYPRO Ruby (BioRad) according to manufacturers’ specifications. The sedimentation analysis of the final eluate of a standard PRC5-PTP purification in a 4 ml linear 10–40% sucrose gradient was carried out as detailed previously (19). For mass spectrometric analysis of proteins associated with PRC5, the gel lane of the final eluate after standard PRC5-PTP purification or after sucrose gradient sedimentation was divided up into even-sized slices and sent to the Keck Biotechnology Resource Laboratory of Yale University (https://medicine.yale.edu/keck/proteomics/) where gel slices were destained and proteins, after reducing them with DTT, were alkylated with iodoacetamide and digested in-gel with trypsin. After peptide extraction, the solution was desalted, and peptides were purified using reverse phase C18 column. Peptides were identified by liquid chromatography/tandem mass spectrometry (LC/MS/MS) in an Elite Orbitrap mass spectrometer. Protein identification was based on MASCOT scores and NCBI non-redundant protein sequence database of eukaryotes with oxidation (M) and propionamide (C) modification. Peptide mass tolerance was set to ± 10ppm and fragment mass tolerance was set to 0.5 Da for analysis of final eluate of standard PRC5-PTP purification or to 0.25 Da after sucrose gradient sedimentation. The mass spectrometry proteomics data has been deposited to the ProteomeXchange Consortium via the PRIDE partner repository with the dataset identifier PXD026239 (37).

To determine the phosphorylation status of RPB1, the largest subunit of RNA polymerase (pol) II, trypanosome extract was electrophoretically separated in an 8% SDS polyacrylamide gel, electroblotted on to PVDF membrane (Millipore Sigma) and detected with a 1:1000 diluted rat immune serum directed against the C-terminal domain of *T. brucei* RPB1 (38), a 1:5000 diluted peroxidase-conjugated goat anti-rat IgG secondary antibody (Thermo Fisher Scientific) and BM chemiluminescence blotting substrate (Roche). For loading controls, RPA1, the largest subunit of RNA pol I, or CRK9 were detected on the same blots with rabbit anti-RPA1 or rat anti-CRK9 immune sera as described previously (39,40).

### RNA immunoprecipitation (RIP)

RIP assays were performed as detailed previously (26). Briefly, PTP-tagged SmD1, PRP19, PRC3 or PRC5 were precipitated from crude trypanosome extract with IgG beads (Millipore Sigma) that bind the ProtA domains of the PTP tag, total RNA was prepared from precipitates using the Trizol reagent, and spliceosomal U snRNAs and SL RNA were detected by primer extension. To quantify the association of PRC5-PTP and PRP19-PTP with U4 snRNA, three independent RIP experiments were conducted for each protein, and the abundance of U4 snRNA relative to that of U2 snRNA in PRC5-PTP and PRP19-PTP precipitates was determined by qPCR of random hexamer-derived cDNA.

## RESULTS

### PRC5 silencing is lethal, causing a pre-mRNA splicing defect

**Figure 1.**
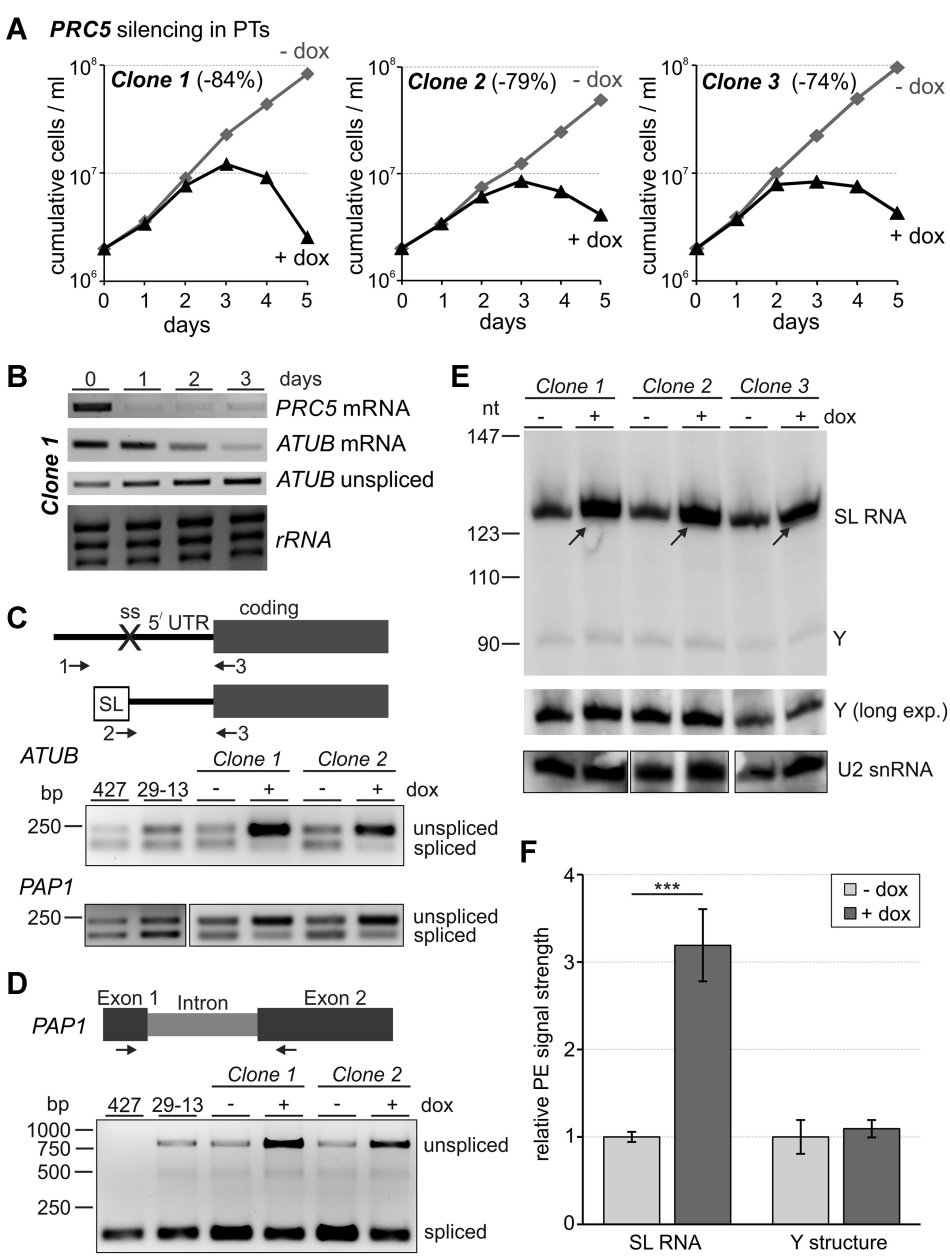
PRC5 is an essential pre-mRNA splicing factor. (**A**) Growth curves of three clonal lines of procyclic trypanosomes (PTs) in the absence and presence of doxycycline (-/+ dox) which, in these lines, triggers *PRC5* silencing on the mRNA level via the RNAi pathway. *PRC5* knockdown efficiency, as indicated in parentheses, was quantified after 1 day of doxycycline treatment by RT-qPCR. (**B**) Semi-quantitative RT-PCR analysis upon doxycycline treatment of *PRC*5 and α-tubulin (*ATUB*) mRNA, and of *ATUB* pre-mRNA that was not *trans*-spliced (unspliced). Ethidium bromide-staining of the large rRNA species indicate comparable RNA preparations for these assays. (**C**) On top, schematic of the 3-primer PCR assay for detection of *trans* splicing defects in random hexamer-derived cDNA. Sense primer 1 locates upstream of the 3’ splice site (ss) and is specific for unspliced pre-mRNA, whereas mRNA-specific sense primer 2 comprises the 20 3’-terminal nucleotides of the spliced leader (SL) and the first five nucleotides of the 5’ UTR. Primer 3 is antisense to the coding region and amplifies cDNA with primers 1 or 2. Below, 3-primer RT-PCR of *ATUB* and *PAP1* RNAs with total RNA preparations of wild-type PTs (strain 427), slower growing 29–13 PTs, and of clones 1 and 2 which were grown in the absence (-) or presence (+) of doxycycline for 3 days. (**D**) On top, schematic of the 2-primer PCR assay to test *cis* splicing of the *PAP1* intron and, on bottom, the corresponding RT-PCR analysis. (**E**) Primer extension of total RNA prepared from clones 1 to 3 with biotinylated oligonucleotides SL_PE and U2_PE which are antisense to SL RNA and U2 snRNA, respectively. Cells were either untreated or induced with doxycycline for 3 days. Arrows point to full-length SL RNA signals that increase upon *PRC5* silencing. The middle panel shows a longer exposure of the specific Y extension product. U2 extension products, shown on the bottom panel, were analyzed on separate gels. (**F**) SL RNA and Y structure primer extension signals were quantified by densitometry and normalized with the U2 signal. Error bars represent one standard deviation, with asterisks indicating a Student's *t* test (two-tailed, unpaired, equal variance) *P* value < 0.001.

PRC5 is a well-conserved protein among trypanosomatids (Supplementary Figure S1) although it appears to be missing in the most divergent trypanosomatid, *Paratrypanosoma confusum*. Accordingly, we could not find sequence similarity to proteins outside of this clade, including the related organism *B. saltans*, which, as trypanosomatids, belongs to the class Kinetoplastea (41). Furthermore, motif and pattern searches as well as protein homology modeling were inconclusive, suggesting that PRC5 is a trypanosomatid-specific protein. To evaluate the gene’s significance for parasite viability and pre-mRNA splicing, we conditionally silenced *PRC5* via RNAi. We integrated plasmid T7-PRC5-stl into the silent spacer of the ribosomal RNA locus in 29–13 procyclic trypanososmes which constitutively express the tetracycline (TET) repressor and T7 RNA polymerase (32). The plasmid contained a TET-regulated T7 promoter to drive expression of a *PRC5*-specific hairpin RNA in the presence of the stable tetracycline derivate doxycycline (dox). We obtained three clonal cell lines, all of which halted growth between 3 and 4 days of dox treatment with a subsequent decline of cell numbers, indicating that *PRC5* is an essential gene (Figure 1A). An initial semiquantitative RT-PCR analysis showed effective depletion of *PRC5* mRNA after 1 day of dox treatment (Figure 1B) and RT-qPCR assays determined that *PRC5* mRNA abundance relative to 18S rRNA at the same time point was reduced by 84%, 79% and 74% for clones 1, 2 and 3, respectively. Moreover, semiquantitative RT-PCR of α tubulin (*ATUB*) RNAs suggested an RNA splicing defect since the abundance of mature *ATUB* mRNA strongly declined over a 3-day time course whereas the level of unspliced *ATUB* pre-mRNA concurrently increased (Figure 1B).

We recently reported 3-primer PCR assays that robustly and efficiently detect SL *trans* splicing defects of *ATUB* and *PAP1* RNAs in cDNA that was reverse-transcribed from total RNA with random hexamers (42). In this assay, primers 1 and 2 are specific for unspliced pre-mRNA and mature mRNA, respectively, because primer 1 anneals upstream of the 3′ splice site and primer 2 comprises the 20 3′-terminal SL nucleotides and the first five nucleotides of the 5′ UTR. Both primers amplify cDNA with antisense primer 3 (Figure 1C). After three days of doxycycline treatment both RNAs exhibited a clear splicing defect because the unspliced product became much stronger and the signal of the spliced product decreased concurrently (Figure 1C). A similar picture was obtained with a 2-primer PCR that spanned the *PAP1* intron, suggesting that *cis*-splicing was impaired too (Figure 1D).

Silencing of splicing factor genes that are essential for the first splicing step has typically resulted in an increase of SL RNA and the disappearance of the Y structure intermediate, the counterpart of the lariat structure in *cis* splicing, over the course of 3 days (19,26,38,43,44). Using a primer extension assay for SL RNA and U2 snRNA, we clearly detected the increase of SL RNA in all three clones upon *PRC5*-silencing (Figure 1E), which was >3-fold higher than in untreated cells when primer extension signals were quantified by densitometry (Figure 1F) or when SL RNA and U2 snRNA were subjected to RT-qPCR analysis (3.16-fold increase on average, standard deviation of 0.12, *P* < 0.001). However, the primer extension assay also showed that the Y structure intermediate, relative to the U2 signal, did not significantly change (Figure 1E and F). This result is in contrast to the complete disappearance of the Y intermediate upon silencing the gene of the PRP19 complex subunit *SPF27* (19) or upon chemical inhibition of analog-sensitive CRK9, a cyclin-dependent kinase whose activity is indispensable for the first splicing step to occur (38,42). In summary, these data identified PRC5 as an essential pre-mRNA splicing factor in trypanosomes. While the increase of SL RNA may indicate that PRC5 has a role in the first splicing step, they also raise the possibility that PRC5 is of more critical importance than PRP19 or CRK9 to the second splicing step, a function which would interfere with the discard of the Y intermediate.

### Tandem affinity purification of PRC5-PTP co-isolated many known splicing proteins

**Figure 2.**
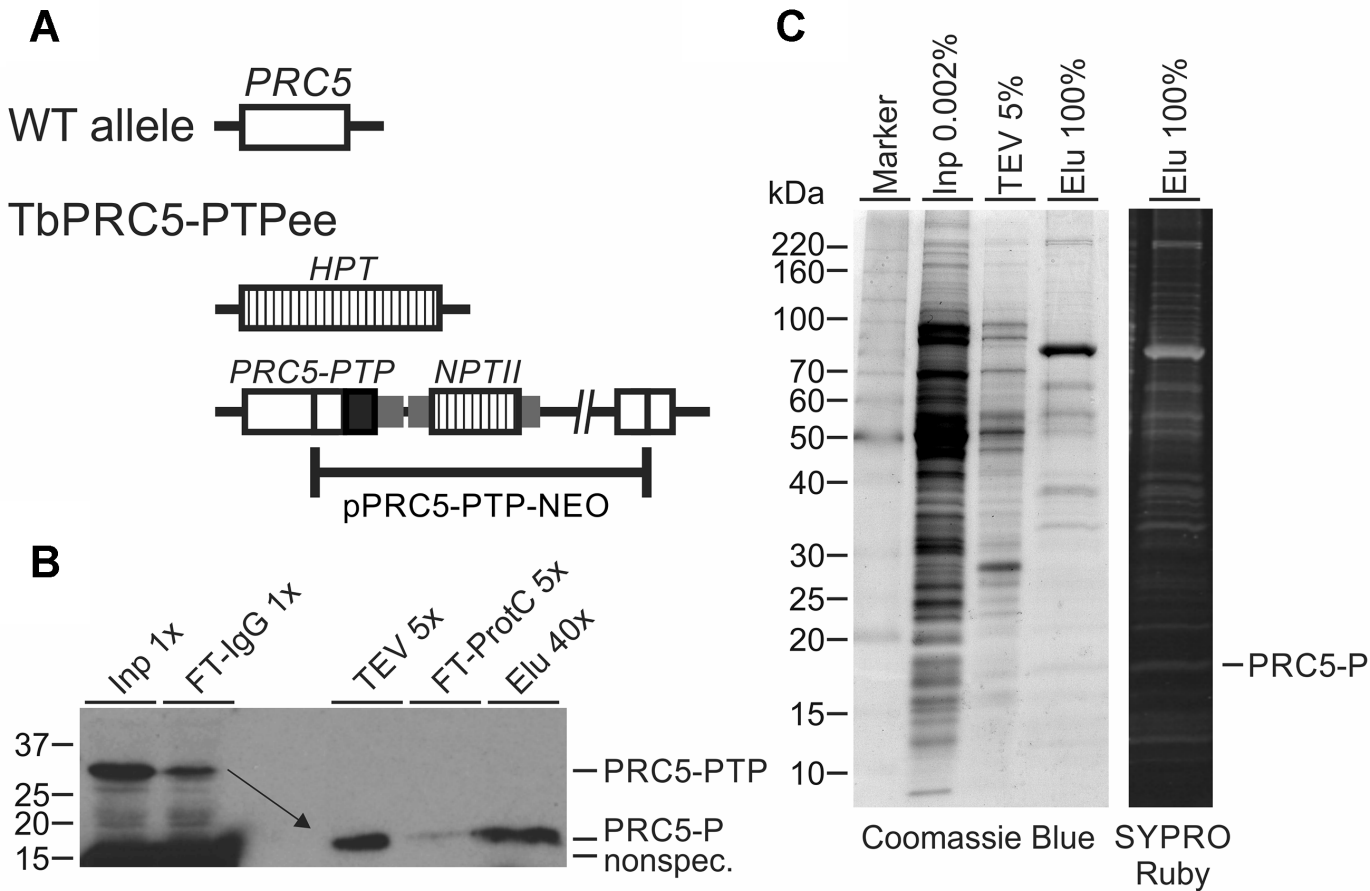
PRC5-PTP tandem affinity purification. (**A**) Schematic (not to scale) of a wild-type (WT) *PRC5* allele and the locus in TbPRC5-PTPee cells in which one allele was knocked out by the coding region of the hygromycin phosphotransferase gene (*HPT*) whereas, in the second allele, site-directed integration of plasmid PRC5-PTP-NEO fused the PTP coding sequence (black box) to the 3’ end of the PRC5 coding region followed by the neomycin phosphotransferase gene II (NPTII). Small gray boxes indicate intergenic regions from the trypanosome genome that provide *trans* splicing and polyadenylation signals for proper RNA processing and striped boxed depict coding regions of the selectable marker genes (**B**) Immunoblot monitoring of PRC5-PTP tandem affinity purification detecting tagged PRC5 with the monoclonal anti-ProtC antibody HPC-4 in crude extract (Inp), the flowthrough of IgG affinity chromatography (FT-IgG), in TEV protease eluate, the flow through of the anti-ProtC immunoaffinity chromatography (FT-ProtC) and the final eluate (Elu). *X*-values indicate relative amounts analyzed. Please note that, as indicated by the arrow, TEV protease cleavage reduces the size of tagged PRC5 by ∼20 kDa. (**C**) Crude extract, TEV eluate and final eluate were separated on a 10–20% SDS polyacrylamide gradient gel and consecutively stained with SYPRO Ruby and Coomassie blue. The percentages specify relative amounts loaded. On the right, the band of tagged PRC5 in the final eluate is identified.

To analyze whether PRC5 is part of a spliceosomal protein complex, we PTP-tagged and tandem affinity-purified the protein. C-terminal tagging was achieved by targeted integration of plasmid PRC5-PTP-NEO into an endogenous *PRC5* allele. In a second transfection, the remaining wild-type allele was replaced by the hygromycin phosphotransferase gene, which resulted in the clonal cell line TbPRC5-PTPee that exclusively expressed PRC5-PTP (Figure 2A). Since these cells did not exhibit a proliferation defect (Supplementary Figure S2) and *PRC5* was shown to be essential for viability (Figure 1A), we concluded that the tag did not impair *PRC5* function.

Immunoblot monitoring of PRC5-PTP TAP revealed the efficiency of each procedural step (Figure 2B). Separation of the final eluate by SDS-PAGE and staining of proteins with Coomassie Blue or Sypro Ruby detected many co-purifying protein bands with particular enrichment of a ∼90 kDa band (Figure 2C). Proteins of the whole lane were identified by liquid chromatography-tandem mass spectrometry (LC/MS/MS). After an early, initial analysis that only detected 21 proteins, two comprehensive analyses were carried out that identified >100 proteins in strongly overlapping data sets (Supplementary Table S2). Overall, 136 proteins were identified in both analyses with MASCOT protein scores >80 (45) and an Expect Value <0.001. Of those, 43 were known splicing factors, 46 highly abundant ribosomal proteins, 25 annotated proteins without known splicing function, 16 ‘sticky’ proteins that have been recurrently purified with other gene expression factors and six proteins with unknown function. To determine whether the latter have similarity with human and yeast splicing proteins, we performed reciprocal BLASTp searches. We found that the protein encoded by gene *Tb927.10.7280* is the homolog of human DHX8 and yeast PRP22, the RNA helicase that is important for release of mRNA from the spliceosome after the second splicing step. Although these DEAH-box RNA helicases generally exhibit a high degree of conservation, NCBI BLASTP of the Tb927.10.7280 amino acid sequence against the human database returned DHX8 on top (*E* = ∼0; 52% identity), a hit backed up by a highly conserved C-terminal sequence motif (Supplementary Figure S3). Fittingly, we found that *Tb927.5.3020* encodes the trypanosome homolog of step 2 factor SLU7 that is involved in the recruitment of PRP22 to the spliceosome. BLASTP of the Tb927.5.3020 amino acid sequence was most similar to human SLU7 (*E* = 4e^–30^, 29% identity) and recognized part of the central SLU7 domain (pfam11708) in the trypanosome protein (aa positions 216–269; *E* = 7.13e^–5^). Correspondingly, *Tb927.5.3020* was the only gene recognized when the trypanosome database was queried with the human SLU7 sequence (*E* = 1e^–25^). Finally, *Tb927.9.11740* encodes the trypanosome homolog of the human B^act^-specific peptidylprolyl isomerase PPIL3, primarily because its enzyme domain (aa 1–169 of 183) is most similar to the ‘Cyclophilin_PPIL3_like’ domain (cd01928, *E* = 2.43e^–67^). After PPIL1, which was identified as a subunit of the trypanosome PRP19 complex (19), PPIL3 is the second such enzyme implicated in trypanosome pre-mRNA splicing. Given that PPIL1 and 3 have no homologs in *S. cerevisiae*, it appears that pre-mRNA splicing in trypanosomes similarly depends on peptidyl-prolyl isomerization as it does in humans. Hence, this finding suggests that *cis*–*trans* proline isomerization of spliceosome components is of ancient evolutionary origin.

On the other hand, we could not identify homologs of the proteins encoded by genes *Tb927.4.3540*, *Tb927.5.990* and *Tb927.5.2910*, which raises the possibility that these are trypanosomatid-specific splicing factors. This notion seems particularly plausible for the *Tb927.4.3540* and *Tb927.5.2910* encoded proteins since they were independently co-purified with PRP19 and SmD1, respectively (19,26). Table 1 lists the splicing factors and new identifications ranked according to their MASCOT scores. Overall, co-purification of more than half of the known splicing proteins confirmed the role of PRC5 in pre-mRNA splicing and suggested that the protein is part of the spliceosome.

**Table 1.** PRC5-PTP co-purified proteins

Rank	MASCOT score	Expect value^1^	Accession number	*Mr* (Da)	Coverage (%)^1^	Annotation	Complex
**1**	**20 000**	**∼0**	**Tb927.10.9660**	**87 636**	**76.6**	**SYF3**	**PRC**
**2**	**18 340**	**∼0**	**Tb927.5.1340**	**92 126**	**71.3**	**SYF1**	**PRC**
3	7632	∼0	Tb927.9.11110	276 796	45.5	PRP8	U5
4	7134	∼0	Tb927.5.2290	249 282	38.8	BRR2 (U5-200K)	U5
**5**	**6648**	**∼0**	**Tb927.2.3400**	**36 571**	**68.3**	**PRC3**	**PRC**
*6*	*4418*	*∼0*	*Tb927.9.10770*	*62 195*	*59.2*	*PABP2*	
**7**	**3743**	**∼0**	**Tb927.8.1930**	**31 680**	**63.9**	**ISY1**	**PRC**
*8*	*3520*	*∼0*	*Tb11.v5.0469*	*49 672*	*38.5*	*Beta tubulin*	
*9*	*3126*	*∼0*	*Tb927.1.2340*	*49 756*	*48.4*	*Alpha tubulin*	
10	2765	∼0	Tb927.10.7280	121 154	45.7	PRP22 * (DHX8)	
11	2727	∼0	Tb927.2.5240	54 200	49.1	PRP19	PRP19
12	2727	∼0	Tb927.11.11610	64 578	45.5	Cactin	
14	2342	∼0	Tb927.11.11330	76 002	49.9	HSP73	
**18**	**1938**	**1.1E^–106^**	**Tb927.11.2960**	**12 026**	**66.8**	**PRC5**	**PRC**
19	1937	2.4E^–122^	Tb927.5.2060	80 135	48.7	CDC5	PRP19
22	1774	4.4E^–112^	Tb92711.10750	66 828	32.4	CWC22	
23	1746	5.1E^–109^	Tb927.10.14360	36 525	44.0	U2A' (U2-40K)	U2
30	1615	3.5E^–76^	Tb927.3.1930	52 847	44.5	PRP17	PRP19
33	1595	1.1E^–91^	Tb927.9.5880	60 845	44.5	SKIP	PRP19
36	1532	2.9E^–86^	Tb927.6.4340	12 784	61.1	SSm2-1 (Sm15K)	U2
39	1461	1.3E^–68^	Tb927.7.6380	23 139	73.7	SSm4	U4
48	1303	9.1E^–61^	Tb927.3.1780	14 027	59.6	LSm8	U6
50	1249	9.5E^–65^	Tb927.5.3020	45 934	53.9	SLU7 *	
54	1157	3.5E^–75^	Tb927.8.5180	13 250	46.5	LSm2	U6
55	1135	1.3E^–56^	Tb927.4.3540	41 174	25.9	Unknown function, conserved	
59	1103	6E^–51^	Tb927.10.11950	22 377	53.6	AD002, CWC15	
61	1084	4.1E^–56^	Tb927.10.10170	48 772	35.6	PRL1	PRP19
63	1060	1E^–58^	Tb927.2.4540	12 324	81.2	SmB	U1, U4, U5, SL
65	1031	2.9E^–53^	Tb927.2.5850	12 481	77.6	SmD2	U1, U2, U4, U5, SL
67	982	7.1E^–52^	Tb927.6.2700	9649	71.8	SmE	U1, U2, U4, U5, SL
69	940	2.9E^–43^	Tb927.11.14150	24 508	43.5	SPF27	PRP19
73	878	8.4E^–40^	Tb927.10.4950	14 732	60.5	SSm2.2 (U2-16.5K)	U2
79	809	2.1E^–36^	Tb927.5.990	21 505	69.1	Unknown function, conserved	
85	706	1.1E^–42^	Tb927.5.2910	19 967	34.2	Unknown function, conserved	
86	676	1.6E^–30^	Tb927.11.13960	14 147	61.4	LSm4	U6
87	662	4.2E^–29^	Tb927.5.4030	10 207	75.4	LSm7	U6
90	624	1.3E^–27^	Tb927.3.3480	13 618	47.7	U2-B^//^	U2
92	607	1.6E^–31^	Tb927.9.11740	20 280	49.4	PPIL3 *	
93	601	2E^–26^	Tb927.9.10250	8356	88.2	SmF	U1, U2, U4, U5, SL
94	597	7.2E^–26^	Tb927.8.2090	21 654	44.2	PPIL1	PRP19
95	587	2.2E^–29^	Tb927.9.3480	20 487	63.9	Cwc21	U5
103	536	2.9E^–30^	Tb927.7.3120	11 714	45.1	SmD1	U1, U2, U4, U5, SL
136	166	1.2E^–4^	Tb927.7380	10 116	56.6	LSm3	U6

List of selected PRC5-PTP co-purified proteins, identified by two independent LC/MS/MS analyses and ranked according to their combined, standardized MASCOT protein scores. The list contains the 12 top-ranked hits including non-spliceosomal proteins (cursive), known splicing factors, newly annotated splicing proteins (marked by an asterisk), and conserved proteins of unknown function. Bold lettering indicates PRC subunits. The complete list of proteins is presented in Supplementary Table S2, comprising, in addition, ribosomal proteins, recurrent contaminants of PTP-purified gene expression factors, and annotated proteins unrelated to pre-mRNA splicing. Moreover, seven co-purified coatomer subunits have been omitted although the coatomer has been implicated in spliceosomal snRNP biogenesis previously (54).

^1^The lower expect value or higher coverage number between the two analyses are given.

### PRC5 is part of a pentameric protein complex of PRP19-related proteins

**Figure 3.**
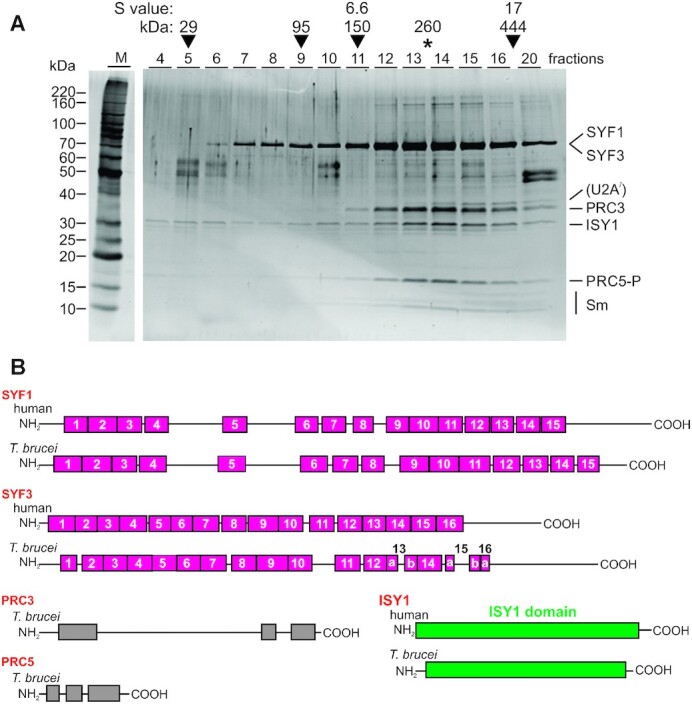
PRC5 is part of a complex with PRP19-related proteins and the trypanosomatid-specific protein PRC3. (**A**) The final eluate of PRC5-PTP tandem affinity purification was sedimented through a 10–40% linear sucrose gradient by ultracentrifugation and fractionated from top to bottom into 20 aliquots. Proteins from each fraction were separated by SDS-PAGE and stained with SYPRO Ruby. Protein bands were excised and identified by LC/MS/MS. For comparison, sedimentations of TEV protease (29 kDa), *Taq* DNA polymerase (95 kDa), IgG (150 kDa, 6.6S) and apoferritin (444 kDa, 17S), were analyzed in parallel gradients (arrowheads). (**B**) Schematic representation to scale of the five PRC subunits. For comparison, the human SYF1, SYF3 and ISY1 homologs are depicted, too. Pink, green and gray boxes indicate TPR domains, ISY1 domains and highly conserved regions among trypanosomatid homologs, respectively.

Among the top PRC5-PTP co-purifying proteins were SYF1, SYF3 and ISY1. All three proteins are part of the yeast NTC whereas, in humans, SYF1 and ISY1 are subunits of the IBC, and SYF3 seems to be independently recruited to the spliceosome (8). This suggested that PRC5 might be in a complex with these ‘PRP19-related’ proteins. To find out, we sedimented the final eluate of a standard PRC5-PTP TAP through a 10–40% linear sucrose gradient by ultracentrifugation, collected 20 fractions from top to bottom and analyzed individual fractions by denaturing SDS-PAGE and SYPRO Ruby staining of proteins (Figure 3A). We detected four protein bands that co-sedimented with a peak in fractions 13 and 14. LC/MS/MS analysis of these bands identified the trypanosome SYF1 and SYF3 homologs in the ∼90 kDa band, PRC3 encoded by gene *Tb927.2.3400* in the 38 kDa band, the ISY1 homolog in the 32 kDa band and PRC5-P in the 18 kDa band. The combined calculated molecular mass of these five proteins is ∼260 kDa which, according to size markers, is in line with a sedimentation peak in fractions 13/14 (Figure 3A). Several bands of minor abundance co-sedimented with these five proteins particularly in higher fractions. Mass spectrometry identified the small proteins as various Sm proteins whereas the band just above PRC3 was identified as the U2 snRNP protein U2A. It therefore appears that some spliceosomal components such as the U2 snRNP remained attached to the PRC throughout sedimentation, dragging the PRC down the gradient and causing the rather broad sedimentation profile. We then repeated TAP with a newly generated cell line expressing PRC3-PTP. As expected, SYF1, SYF3, ISY1 and PRC5 co-purified with PRC3-PTP, which confirmed the presence of the PRC (Supplementary Figure S4A). However, the complex was dissociated after sedimentation through a sucrose gradient, indicating that the large PTP tag on PRC3 destabilized the PRC (Supplementary Figure S4B).

In a first bioinformatics analysis of PRC subunits, we determined their domain structures (Figure 3B). SYF1 and SYF3 are elongated α-helical proteins characterized by 15 and 16 tetratricopeptide repeats (TPRs), respectively. Multiple sequence alignment showed that these TPRs are conserved in trypanosomatid SYF1 (Supplementary Figure S5A); although there is sequence deviation, TPR key residues are often present and secondary structure predication is largely in accordance with human and trypanosome SYF1. The same is true for SYF3 although the sequence alignment suggests that in trypanosomatid SYF3 TPR-13 and -15 are interrupted by sequence insertions and only the N-terminal half of TPR-16 has been conserved (Supplementary Figure S5B). Nearly all human ISY1 (aa 1–266 out of 285 aa) is defined as ISY1 domain (accession pfam06246), and this domain is conserved almost in its entirety in the trypanosome homolog (aa 13–255, *E* = 2.37e^–33^). PRC3 and PRC5 each possess three well-conserved domains among trypanosomatids (Supplementary Figures S1 and S6). However, these domains do not exhibit similarity to established protein domains, and BLASTp searches of human and yeast databases did not return homologs. Moreover, PRC3 and PRC5 seem to be confined to trypanosomatids, while SYF1, SYF3 and ISY1 have clear homologs in the related kinetoplastid organism *B. saltans* (accession numbers BSAL_32920, BSAL_27400 and BSAL_05560, respectively), homologs of PRC3 and PRC5 could not be identified.

### PRP19 and PRC are distinct complexes in trypanosomes

**Figure 4.**
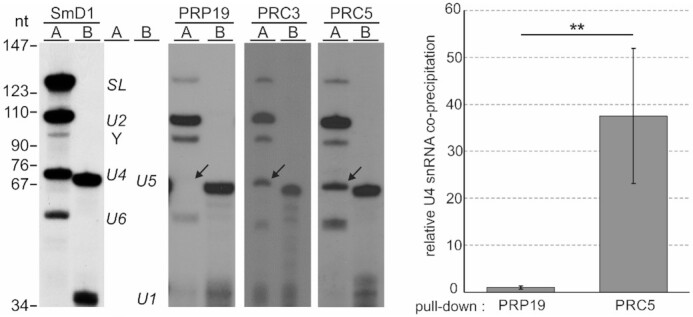
PRC is associated with U2, U4, U5 and U6 snRNA. Extracts were prepared from procylic cell lines that express SmD1, PRP19, PRC3 or PRC5 with a C-terminal PTP tag, and PTP-tagged proteins were pulled down with IgG beads that bind ProtA. Total RNA was prepared from precipitates and analyzed by two primer extension reactions. Reaction A contained four radiolabeled DNA oligonucleotides that are complementary to SL RNA and to U2, U4 and U6 snRNA, and reaction B was conducted with U1- and U5-specific oligonucleotides. The SmD1 pull-down served as a positive control, and it should be noted that we have previously shown that none of the snRNAs co-precipitated with the DNA replication protein ORC1 that was equivalently PTP-tagged (19). Arrows point to the U4-specific extension product. The bar diagram on the right shows the amount of co-precipitated U4 snRNA relative to U2 snRNA, as determined by RT-qPCR, between PRP19 and PRC5 pull-downs with three independent extract preparations. Error bars represent one standard deviation, with asterisks indicating a Student’s *t* test (two-tailed, unpaired, unequal variance) *P* value of < 0.01.

Since the yeast NTC combines PRP19 complex and PRP19-related proteins in one stable complex, it was possible that PRC and PRP19 complex are subcomplexes of a trypanosome NTC that fell apart during extract preparation or tandem affinity purification. In support of this possibility, all PRC subunits were copurified with PRP19-PTP (19) and all subunits of the PRP19 complex copurified with PRC5-PTP (Table 1). However, we noted that SSm4, a U4-specific Sm protein in trypanosomes (46), was among the top Sm proteins that co-purified with PRC5 (Table 1), suggesting that the PRC is recruited into the spliceosome before the release of the U4 snRNP in the transition from B to B^act^. Conversely, RNA immunoprecipitation of PRP19, CDC5 and SPF27, all subunits of the PRP19 complex, contained U2, U5 and U6 snRNA but only traces of U4 snRNA which indicated that, in trypanosomes, the PRP19 complex is stably recruited during activation of the spliceosome (19). If this was true, then PRC and PRP19 complex would be distinct entities that are recruited into the spliceosome separately at different time points of the splicing cycle. To test this, we precipitated PRC5-PTP from extract of TbPRC5ee cells, prepared total RNA from the precipitate and assayed for all five spliceosomal U snRNAs and the SL RNA in two established primer extension reactions (26). In reaction A, oligonucleotides complementary to U2, U4 and U6 snRNA as well as the SL RNA were used, whereas reaction B contained oligonucleotides specific for U5 and U1 snRNA. In a positive control precipitation of the common snRNP protein SmD1 that was equivalently PTP-tagged in a separate cell line as PRC5 in TbPRC5ee cells, the pellet contained all five spliceosomal snRNAs and the SL RNA including the Y structure (Figure 4). Pull-down of PRC5 efficiently co-precipitated U2, U4, U5 and U6 snRNAs, whereas the U1 snRNA was hardly detectable. To confirm this result, we repeated the experiment with PRC3-PTP-expressing cells and saw again that the pull-down of PRC3-PTP co-precipitated U4 snRNA whereas a repeat of the PRP19-PTP pull-down confirmed the lack of U4 snRNA in these assays. Quantification of U4 snRNA abundance relative to the U2 snRNA level by RT-qPCR revealed that U4 co-precipitation in PRC5-PTP versus PRP19-PTP pellets was significantly higher (Figure 4, right panel). We therefore concluded that PRC and PRP19 complex are distinct entities that are recruited into the spliceosome consecutively.

### PRC3 is particularly important for pre-mRNA splicing in bloodstream trypanosomes

**Figure 5.**
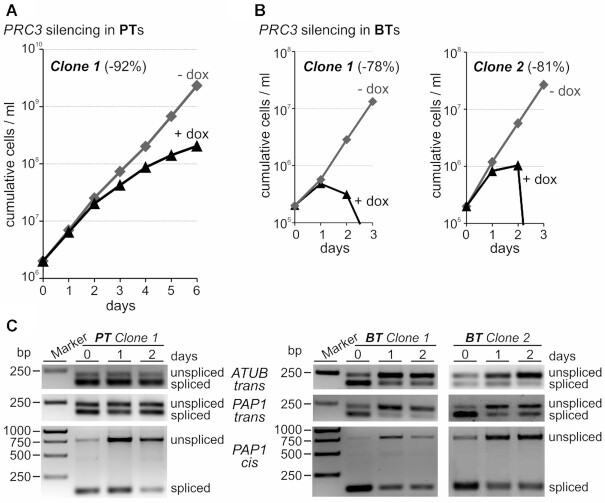
PRC3 is an essential splicing factor in bloodstream *T. brucei*. (**A**) On top, culture growth of a representative procyclic trypanosome (PT) line over 6 days in which doxycycline reduced *PRC3* mRNA by 92% after one day of induction. (**B**) Comparable analysis of two bloodstream trypanosome (BT) cell lines in which doxycycline reduced *PRC3* mRNA by 78% and 81% after one day. (**C**) 3- and 2-primer RT-PCR assays to determine *trans* splicing defects of *ATUB* and *PAP1* RNAs, and *cis* splicing defects of *PAP1* RNA, respectively.

*PRC3* appears to be a trypanosomatid-specific gene because, like *PRC5*, it is present in all trypanosomatids for which genome data are available, whereas a homologous gene could not be found in *B. saltans* or other eukaryotes. To investigate whether this gene is essential for parasite viability and pre-mRNA splicing, we generated clonal cell lines for conditional *PRC3* silencing in procyclic trypanosomes. In contrast to the *PRC5* results (see Figure 1), effective knockdown of *PRC3* did not halt culture growth, although it did affect growth over the six-day time course (Figure 5A and Supplementary Figure S7). According to the rather mild growth defect, a slight effect on splicing was detected in the *PAP1 cis* splicing assay only, suggesting that PRC3 has an accessory, stabilizing role in splicing (Figure 5A and Supplementary S7B). Since bloodstream trypanosomes are kept at a higher temperature than procyclics (37 versus 28°C), and it was previously shown that differential pseudouridylation of snRNAs between the two life cycle stages strengthened spliceosomal RNA–RNA and RNA–protein interactions in bloodstream trypanosomes (47), it was possible that PRC3′s splicing function is more important in the latter. To find out, we generated and analyzed two bloodstream trypanosome lines for conditional *PRC3* silencing. Both cell lines were highly sensitive to doxycycline, stopped growing one day after induction of the *PRC3* knockdown, and rapidly died between days 2 and 3. Moreover, *trans* and *cis* splicing defects became readily apparent on day 1 and 2, strongly indicating that PRC3 is an essential splicing factor in this life cycle stage of the parasite (Figure 5B). To exclude the possibility that PRC3 and PRC5 swap an essential function in the two life cycle stages, we generated two clonal cell lines for conditional silencing of *PRC5* in the bloodstream stage. Similar to the *PRC3* knockdown, *PRC5*-silenced cells ceased growth and exhibited clear splicing defects after one day of induction, indicating that PRC5 is an essential splicing gene in both life cycle stages (Supplementary Figure S8).

### Apparent cross-talk between pre-mRNA processing and RNA pol II in trypanosomes

In trypanosomes, the activity of cyclin-dependent kinase CRK9 is a pre-requisite for pre-mRNA splicing to occur. However, *CRK9* silencing or chemical inhibition of analog-sensitive CRK9 also resulted in loss of phosphorylation of RPB1, the largest subunit of RNA pol II (38,42). While the carboxy-terminal domain (CTD) of trypanosome RPB1 is special since it does not contain the heptad repeats whose phosphorylation status directs the transcription cycle in other eukaryotes, it does carry up to 17 phospho-sites (48,49). Hence, RPB1 phosphorylation may be specifically linked to pre-mRNA splicing in trypanosomes. Having generated *PRC* knockdown cell lines, we wished to test this hypothesis.

**Figure 6.**
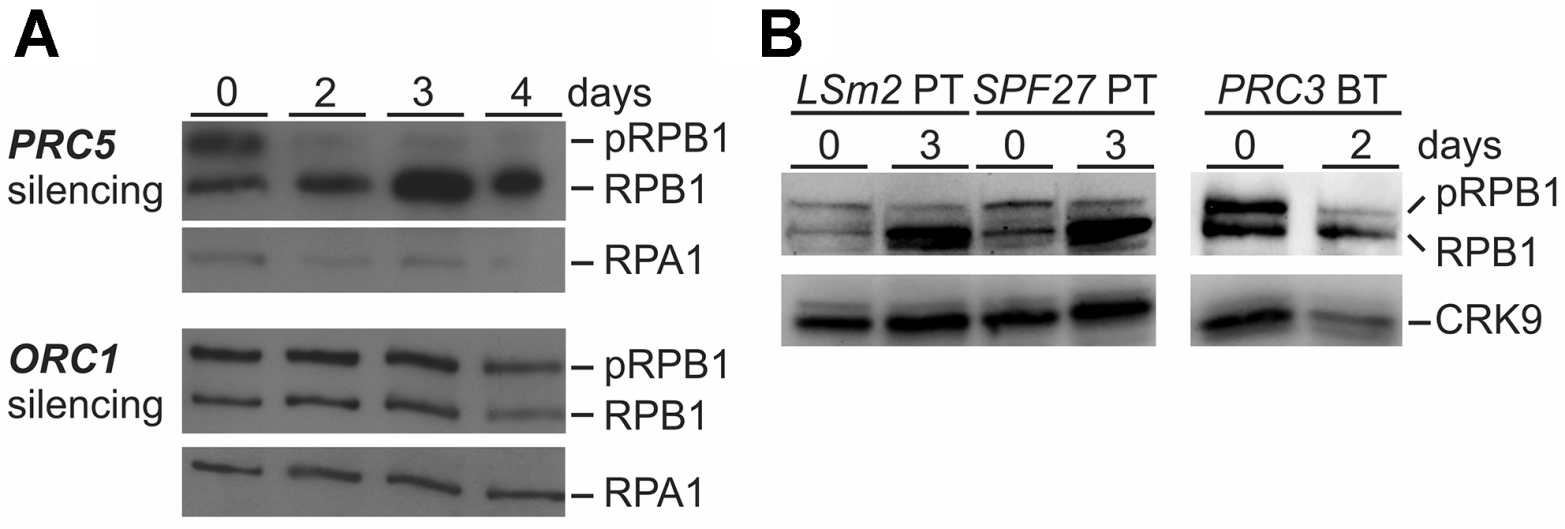
Block of pre-mRNA splicing leads to loss of RPB1 phosphorylation. (**A**) Immunoblot of whole cell lysates prepared from procyclic trypanosomes (PT) in which *PRC5* or *ORC1* were silenced for up to 4 days, detecting phosphorylated (pRPB1) or unphosphorylated RPB1 with a polyclonal immune serum that was raised against the CTD of the protein. RPA1 was detected as a loading control on the same blot (**B**) Corresponding analysis of procyclics in which *LSm2* and *SPF27* were silenced for 3 days, and of a bloodstream trypanosome (BT) line in which *PRC3* was silenced for 2 days. CRK9 was detected as a loading control.

By generating an anti-RPB1 immune serum, we demonstrated previously by immunoblotting that RPB1 was present in two major bands, the upper of which could be resolved into the lower band by alkaline phosphatase (38). In these assays, loss of the upper band resulted in a stronger lower band than the combined strength of both bands in untreated extract which likely is due to the fact that the immune serum was raised against the unphosphorylated CTD that was expressed in and purified from bacteria (38). Accordingly, *PRC5* silencing effectively reduced the phosphorylated RPB1 band whereas the signal strength of the lower band was strongly increased (Figure 6A). Since we previously established a procyclic cell line for conditional silencing of the DNA replication gene *ORC1* and showed that its knockdown resulted in a similar growth defect as the silencing of splicing genes (19), we analyzed RPB1 in *ORC1*-silenced cells and found no effect on the distribution of the two RPB1 bands (Figure 6A). We then revived and analyzed already characterized procyclic cell lines for silencing of *LSm2*, an essential component of the U6 snRNP and of *SPF27*. In addition, we silenced *PRC3* in bloodstream trypanosomes. In each case, the signal of the lower band relative to the upper band became dominant upon silencing (Figure 6B). Together, these results confirm our notion that blocking the splicing process specifically leads to a loss of RPB1 phosphorylation, which suggests a mechanistic link between these two gene expression pathways.

## DISCUSSION

Within the overarching goal to comprehensively characterize the splicing factor repertoire in the evolutionary divergent parasite *T. brucei*, we focused on a protein of unknown function, PRC5, that had been co-purified with two other splicing proteins previously. Conditional gene silencing experiments demonstrated that PRC5 is an essential factor for pre-mRNA splicing and cell viability. Moreover, TAP combined with sucrose gradient sedimentation analysis revealed that PRC5 occurs in a complex with homologs of the three PRP19-related proteins SYF1, SYF3 and ISY1, as well as with PRC3, an apparent second trypanosomatid-specific protein. While PRC5 proved to be an essential splicing protein in both life cycle stages, PRC3 appears to have a supporting function that is indispensable in the mammalian-infective stage of the parasite.

**Figure 7.**
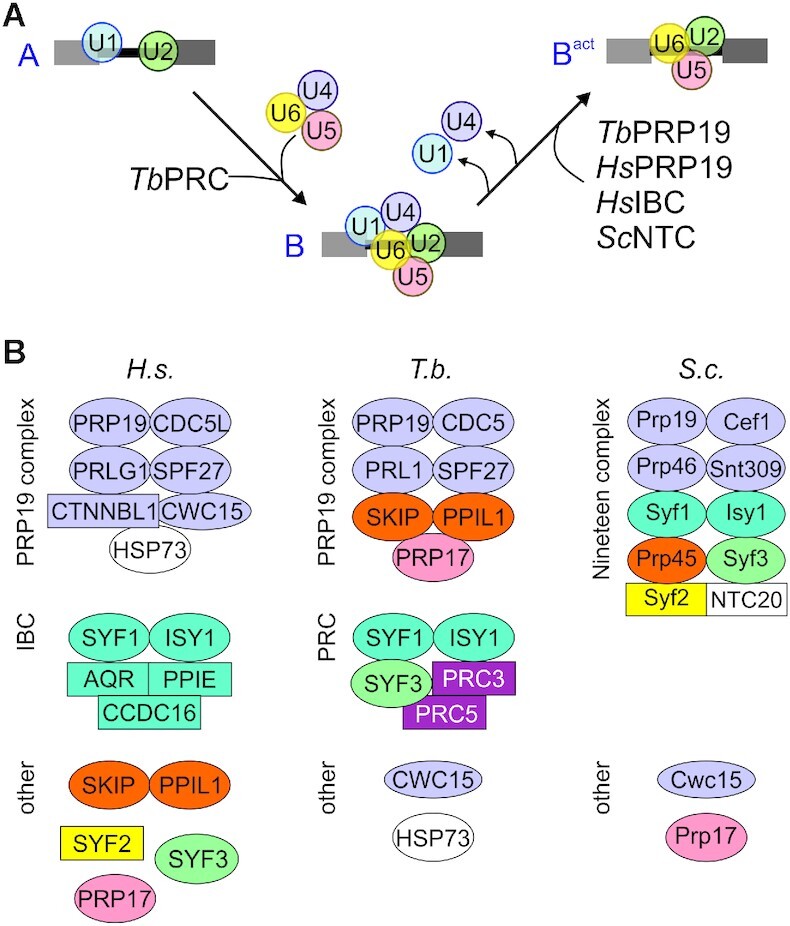
Recruitment and pre-organization of PRP19-associated proteins in humans, *S. cerevisiae* and *T. brucei*. (**A**) Model of stable recruitment of preassembled PRP19 and PRP19-related complexes during assembly from A to B to activated (B^act^) spliceosome in humans (*Hs*), yeast (*Sc*) and trypanosomes. *Tb*PRC is associated with U2, U4, U5 and U6 snRNA whereas the *Tb*PRP19 complex does not co-precipitate U4 snRNA, suggesting that *Tb*PRC is stably recruited into the spliceosome prior to or during B complex formation whereas *Tb*PRP19, as *Hs*PRP19, *Hs*IBC and *Sc*NTC, becomes stably integrated into the spliceosome during the formation of the activated B complex (B^act^). (**B**) Pre-assembled complexes of PRP19-associated proteins. In humans, PRP19 complex (purple subunits) and Intron Binding Complex (IBC, turquoise subunits) form before they are recruited into the spliceosome whereas in yeast a large NTC is assembled. In *T. brucei*, PRP19 and PRC form distinct complexes. However, the subunit composition differs among all complexes in these three organisms. Circles indicate subunits that are conserved between human/yeast and trypanosomes whereas rectangles indicate subunits that are either not present in or specific to trypanosomes. Please note the different yeast/human nomenclatures for CDC5L/Cef1, PRLG1/PRP46, SPF27/Snt309 and SKIP/Prp45.

In yeast, SYF1, SYF3 and ISY1 are assembled in the large NTC which is stably recruited into the spliceosome before the first splicing step (17,20,50), raising the possibility that trypanosome PRC and PRP19 complexes are part of a larger NTC-like complex that may have dissociated during extract preparation. However, our finding that PRC subunits, in contrast to those of the PRP19 complex, are stably associated with U4-containing splicing complexes argues against this notion and strongly indicates that PRC is distinct from the PRP19 complex and recruited into the spliceosome prior to PRP19 when the B complex is still intact. This property distinguishes the PRC from PRP19-related proteins in humans and yeast, all of which become stably associated during activation of the spliceosome (Figure 7A). Despite this difference, it appears that, like in the human system, PRP19-associated proteins in trypanosomes form two distinct non-snRNP protein complexes and the PRC is the counterpart of human IBC. Accordingly, trypanosome PRC and human IBC are both composed of five subunits and share SYF1 and ISY1 (Figure 7B). Besides these two subunits, human IBC consists of the RNA helicase Aquarius (AQR), peptidylprolyl isomerase E (PPIE) and the coiled-coil protein CCDC16 (15). None of these three subunits is present in PRC which harbors the conserved subunit SYF3 that, in human cells, appears to be recruited independently of a protein complex (8) as well as PRC3 and PRC5 which do not contain highly conserved RNA helicase, ATP-binding or peptidylprolyl isomerase domains. In addition, PRC3 and PRC5 do not share recognizable sequence homology to CCDC16. Correspondingly, AQR, PPIE and CCDC16 have no homologs in yeast, and there seems to be no AQR homolog in kinetoplastid organisms. So far, it has been established that the ATPase activity of AQR is essential for the first splicing step whereas it remains to be shown whether AQR helicase activity is required for the splicing process (15). While the lack of AQR suggests that splicing in trypanosomes and yeast functions without this additional ATPase requirement, it is possible that PRC and IBC interact with the spliceosome in similar fashion. Cross-linking experiments revealed multiple interactions between IBC subunits, including SYF1 and ISY1, and the U2-associated complexes SF3A and SF3B (15). In trypanosomes both complexes were biochemically characterized and all ten subunits unambiguously identified (1,51–53). Interestingly though, with the exception of SF3B3 (aka SF3B130), which was detected in tandem affinity purification of SmD1 (26), these proteins did not co-purify with other splicing factors including PRC, suggesting that spliceosomal interactions with these two complexes are either not stable or too transient in trypanosome extracts to be readily detected.

Overall, it appears that proteins associated or complexed with PRP19 are differently organized among the human, yeast and trypanosome systems (Figure 7B). What is common is the core complex of PRP19, CDC5, PRL1 and SPF27 that is present in the human and trypanosome PRP19 complex and the yeast NTC (14,16,19,20). Other subunits vary. For example, human SKIP appears to be recruited to the spliceosome independent from PRP19 complex or IBC (8), whereas it appears to be a *bona fide* subunit of the trypanosome PRP19 complex and the yeast NTC where it is termed PRP45. Another example is SYF3 that is an independent protein in humans, a PRC subunit in trypanosomes and part of the NTC in yeast. Interestingly, while trypanosomes possess several homologs of human splicing factors that are not present in yeast such as PPIL1, SF3B6 and U5-40K (19,26,51,52,54), there seems to be no homolog of SYF2 which is present in both humans and yeast. In yeast, Syf2 is recruited to the spliceosome as an NTC subunit, stabilizing the U2/U6 helix II in the activated spliceosome (21,22), whereas in humans, SYF2 is recruited independently into the C spliceosome where it binds the rearranged U2/U6 helix II. Although it is likely that trypanosomes form corresponding U2/U6 helices, trypanosomes either do not need the stabilizing role of SYF2 or they use a trypanosome-specific protein for this task.

Since trypanosomes use the spliceosome almost exclusively for SL *trans* splicing, PRC5 and PRC3 may be *trans* splicing-specific factors. Indeed, two nematode-specific splicing proteins, SL175 and SL30, were found to be important for SL *trans* splicing but not for intron removal in *Ascaris lumbricoides*, linking the 5' splice site on the SL RNA to the branch point on pre-mRNA through interaction with the branch point binding protein SF1 (55). However, PRC5 and PRC3 seem to be general splicing factors. After knockdown of either gene, the 2-primer PCR assay around the *PAP1* intron sequence suggested that both genes are important for intron removal. Although it is possible that a specific block of *trans* splicing disturbs the ratio of *PAP1* mature and pre-mRNA indirectly, for example unspliced pre-mRNA in the nucleus may be more stable than mature mRNA in the cytoplasm, this scenario is unlikely. We found that about one third of *trans*-spliced and polyadenylated *PAP1* RNA retains the intron and remains in the nucleus in unperturbed cells (A. Srivastava & A. Günzl, unpublished data). Hence, a specific block of *trans* splicing should lead to conversion of nuclear, intron-containing *PAP1* RNA to mature mRNA which we did not observe.

Having generated conditional knockdown cell lines for essential splicing genes, we also investigated whether there is a link between pre-mRNA splicing and RPB1 CTD phosphorylation in trypanosomes. Silencing of these genes resulted in loss of CTD phosphorylation, suggesting functional cross-talk between RNA pol II transcription and pre-mRNA processing. This finding was unexpected because, due to post-transcriptional mRNA capping by SL *trans* splicing, trypanosomes can utilize other RNA pols than RNA pol II, namely RNA pol I or bacteriophage RNA pol, to produce functional mRNA (33,56). However, transcription by these RNA pols is considerably stronger than that of RNA pol II, bacteriophage RNA pols have been tested only for individual reporter genes, and RNA pol I transcription of protein coding genes appears to be spatially linked to a potential splicing compartment where SL RNA is made (57). Hence, it is possible that, as in other systems, a phosphorylated CTD mediates interaction between RNA pol II and the splicing machinery, enabling efficient co-transcriptional processing of pre-mRNA along the directional gene arrays, whereas disruption of this interaction causes the observed phosphorylation loss. In accordance with this notion, ChIP-seq of RNA pol II revealed that the enzyme pauses downstream of SL addition sites in 5′-terminal coding regions, presumably facilitating co-transcriptional pre-mRNA processing (58).

Trypanosomatids, as members of the newly named clade Discoba, have phylogenetically diverged early from the Opisthokonta, the common branch of humans and yeast (59). Hence, parallels that can be drawn from trypanosomes to the yeast and human systems are likely of ancient evolutionary origin. As this study indicates *cis–trans* proline isomerization may have been important for spliceosome function early on. On the other hand, essential and deviating or even unique aspects of the trypanosome spliceosome may be exploited to chemotherapeutically target the parasite. The use of benzoxaraboles as anti-parasitic compounds have validated pre-mRNA processing in trypanosomes and other parasites as suitable drug targets (60–62). Correspondingly, PRC5 and PRC3 are promising target candidates. Therefore, the next step has to be the analysis of these proteins’ specific functions, their interacting protein domains and their direct binding partners.

## Supplementary Material

gkab1152_Supplemental_Files

## Notes

Present address: Monika Tasak, Department of Biochemistry and Biophysics, Center for RNA Biology, University of Rochester School of Medicine and Dentistry, 601 Elmwood Avenue, Rochester, NY 14642, USA.
