# Supplementary material for: A distinct complex of PRP19-related and trypanosomatid-specific proteins is required for pre-mRNA splicing in trypanosomes
Source: Nucleic Acids Res. 2021 Dec 1;49(22):12929–42. doi: 10.1093/nar/gkab1152 (PMC8682746; doi:10.1093/nar/gkab1152)
Supplement: gkab1152_Supplemental_Files [file gkab1152_supplemental_files.zip › supplement.pdf]

Srivastava *et al.* – Supplemental material

Table S1 List of DNA oligonucleotides used in RNA analysis.

Table S2 Mass spectometric identification of PRC5-PTP co-purified proteins.

Figure S1 PRC5 is conserved among trypanosomatids.

Figure S2 C-terminal PTP-tagging of PRC5 is functional.

Figure S3 The C-terminal amino acid sequences of human and trypanosome DHX8/PRP22 are similar.

Figure S4 Tandem affinity purification of PRC3-PTP.

Figure S5 Conservation of the TPR structure in trypanosomatid SYF1 and SYF3 homologs.

Figure S6 PRC3 is conserved among trypanosomatids in its $5^{\prime}$- and $3^{\prime}$-terminal regions.

Figure S7 Analysis of clonal procylic trypanosome (PT) lines in which *PRC3* was conditionally silenced.

Figure S8 Analysis of clonal bloodstream trypanosome (BT) lines in which *PRC5* was conditionally silenced.

Supplemental References

Table S1. List of DNA oligonucleotides used in RNA analysis

RNA	Name	Usage	Sequence (5′ - 3′)
PRC5	0465RNAiFqPCR	RT-(sq, q)PCR	GTAGTGAGCACCACCCTTGA
	0465RNAiRqPCR	RT-(sq, q)PCR	ATCCTTTGCTGGTGGGACTT
PRC3	3400qPCRRNAiF	RT-(q)PCR	GTGCTACGGATCTGATGTTT
	3400qPCRRNAiR	RT-(q)PCR	TCTATTGACCGCAGAAGTG
PAP1	SLsense PAP1	3-primer *trans* splicing assay	ACAGTTTCTGATCTATATTGGAAGA
	PAP PremRsen1	3-primer *trans* splicing assay	TGATGCACCTTTTTATGTGCTCT
	PAP1 Exon1 Rev	3-primer *trans* splicing assay	AATGGAAGGGCATTCGGCCAACA
	Exon 1 Fw	2-primer *cis* splicing assay	GTGCAGCGGCACTCCCAAAAC
	Exon 2 Rv	2-primer *cis* splicing assay	CGTTAAAACAGATGGACAAATC
α tubulin (*ATUB*)	ATUB Fw q3	RT-(sq, q)PCR of *ATUB* mRNA	GTGCATTGAACGTGGATCTG
	ATUB Rv q3	RT-(sq, q)PCR of *ATUB* mRNA	GAGAGTTGCTCGTGGTAGGC
	ATUB Fw q1	RT-(sq, q)PCR of *ATUB* pre-mRNA	GTAAGTGGTGGTGGCGTAAG
	ATUB Rv q1	RT-(sq, q)PCR of *ATUB* pre-mRNA	CAATGTGGATGCAGATAGCC
	SLsense ATUB	3-primer *trans* splicing assay	ACAGTTTCTGATCTATATTGATCTT
	ATUB Fw q1	3-primer *trans* splicing assay	GTAAGTGGTGGTGGCGTAAG
	ATUB Rv q1	3-primer *trans* splicing assay	CAATGTGGATGCAGATAGCC
18S rRNA	18SrRNAcoding5′	RT-qPCR	TCATCAAACTGTGCCGATTAC
	18SrRNAcoding3′	RT-qPCR	CTATTGAAGCAATATCGG
SL RNA	SL_PE	RT-qPCR, Primer extension	CGACCCCACCTTCCAGATTC
	SLsense	RT-qPCR	ACAGTTTCTGTACTATATTG
	bio-SL_PE	Primer extension	Biotin-CGACCCCACCTTCCAGATTC
U2 snRNA	U2_PE	RT-qPCR, Primer extension	ACAGGCAACAGTTTTGATCC
	U2 5’	RT-qPCR	ATATCTTCTCGGCTATTTAGC
	bio-U2_PE	Primer extension	Biotin-ACAGGCAACAGTTTTGATCC
U1 snRNA	U1_PE	Primer extension	AGCACGGCGCTTTCGTGATG
U4 snRNA	U4_PE	Primer extension	TACCGGATATAGTATTGCAC
U5 snRNA	U5_PE	Primer extension	CCGCTCGAGGACACCCCAAAGTTT
U6 snRNA	U6_PE	Primer extension	GGGAGAGTGCTAATCTTCTC

Table S2: Mass spectometric identification of PRC5-PTP co-purified proteins. Listed are all proteins that were identified with a MASCOT protein score >80 and an Expect value of E < 0.001 in both mass spec 1 and 2

Color code - **Blue** : PRC subunits, **Yellow**: PRP19 complex subunits, **Orange**: splicing factors, **Pink:** newly annotated splicing factors or proteins of unknown function, **No Highlighting**: unrelated proteins, **Green**: ribosomal proteins, **Gray**: sticky proteins (recurrent TAP co-purifiers)

Rank - The overall rank was determined by the sum of the standardized protein scores of mass specs 1 & 2 (see columns T to V)

	**mass spec 1**								**mass spec 2**				**mass spec 3** (Mascot Score >30)					
**Rank**	**MASCOT Score**	**Score Rank**	**Expectation**	**% Coverage**	**emPAI**	***Mr* (Da)**	**Protein ID**	**Protein Name / Annotation**	**MASCOT Score**	**Score Rank**	**Expectation**	**% Coverage**	**MASCOT Score**	**Score Rank**	**% Coverage**	**std. score mass spec 1**	**std. score mass spec 2**	**std. score 1 & 2**
**1**	12,809	1	0	76.6	16.03	87,636	Tb927.10.9660 / Tb427.10.9660	SYF3	8,048	1	0	72.8	2497	3	29.0	10,000	10,000	20,000
**2**	11,270	2	0	71.3	19.37	92,126	Tb927.5.1340 / Tb427.05.1340	SYF1	7,679	2	0	71.3	819	6	18.8	8,799	9,542	18,340
**3**	3,955	5	0	45.5	2.48	276,796	Tb927.9.11110 / Tb427tmp.211.2420	PRP8 [U5]	3,657	3	0	37.9				3,088	4,544	7,632
**4**	3,980	4	0	38.8	2.79	249,282	Tb927.5.2290 / Tb427.05.2290	BRR2 (U5-200K) [U5]	3,241	4	0	34.7				3,107	4,027	7,134
**5**	4,675	3	0	68.3	15.79	36,571	Tb927.2.3400 / Tb427.02.3400	PRC3	2,413	5	0	65.9	3135	2	49.4	3,650	2,998	6,648
**6**	3,393	6	0	59.2	5.45	62,195	Tb927.9.10770 / Tb427tmp.211.2150	PABP2	1,424	7	0	46.3	166	12	7.0	2,649	1,769	4,418
**7**	2,369	8	0	63.9	18.92	31,680	Tb927.8.1930 / Tb427.08.1930	ISY1	1,524	6	0	63.9	3804	1	57.2	1,849	1,894	3,743
**8**	2,948	7	0	38.5	3.86	49,672	Tb11.v5.0469	beta tubulin	981	13	1.60E-94	31.7	833	5	20.6	2,302	1,219	3,520
**9**	2,104	9	0	48.4	4.27	49756	Tb927.1.2340 / Tb427.01.2340	alpha tubulin	1,194	8	7.60E-116	46.4	366	9	10.9	1,643	1,484	3,126
**10**	1,749	12	0	45.7	2.68	121,154	Tb927.10.7280 / Tb427.10.7280	PRP22 (DHX8), RNA helicase	1,126	11	5.40E-109	32.0				1,365	1,399	2,765
**11**	1,676	13	0	49.1	2.66	54,200	Tb927.2.5240 / Tb427.02.5240	PRP19 [PRP19 complex]	1,142	10	1.30E-110	46.3				1,308	1,419	2,727
**12**	1,601	14	0	45.5	2.60	64,578	Tb927.11.11610 / Tb427tmp.01.3480	Cactin	1,189	9	2.50E-115	37.6				1,250	1,477	2,727
**13**	2,000	11	0	45.5	2.54	49,105	Tb927.10.2100 / Tb11.v5.1046	elongation factor 1- alpha	633	39	9.40E-60	32.9	78	14	12.2	1,561	787	2,348
**14**	2,028	10	0	49.9	2.50	76,002	Tb927.11.11330 / Tb427tmp.01.3110	HSP73	611	42	1.60E-57	28.2				1,583	759	2,342
**15**	1,417	17	0	29.5	1.18	132,854	Tb927.4.450 / Tb427.04.450	coatamer alpha	970	15	2.30E-93	21.4				1,106	1,205	2,312
**16**	1,058	23	3.00E-102	68.6	27.70	30,625	Tb11.v5.1059	40S ribosomal protein S4, putative	958	16	3.40E-92	61.4				826	1,190	2,016
**17**	978	28	3.30E-94	52.0	9.08	41,045	Tb11.v5.0531	fructose-bisphosphate aldolase, glycosomal	973	14	1.10E-93	49.6				764	1,209	1,973
**18**	1,103	22	1.10E-106	66.8	39.87	12,026	Tb927.11.2960 / Tb427tmp.02.0465	PRC5	867	18	4.20E-83	66.8	1205	4	53.7	861	1,077	1,938
**19**	1,259	18	2.40E-122	48.7	1.96	80,135	Tb927.5.2060 / Tb427.05.2060	CDC5 [PRP19 complex]	768	26	3.20E-73	31.3				983	954	1,937
**20**	1,215	19	6.50E-118	36.6	1.41	94,485	Tb927.2.6050 / Tb427.02.6050	beta prime COP protein	773	25	9.80E-74	28.0				949	960	1,909
**21**	1,543	16	0	54.1	2.47	46,591	Tb11.v5.0650	enolase	534	52	9.00E-50	39.2				1,205	664	1,868
**22**	1,157	20	4.40E-112	32.4	1.69	66,828	Tb927.11.10750 / Tb427tmp.01.2520	CWC22	701	29	1.60E-66	29.3				903	871	1,774
**23**	1,126	21	5.10E-109	44.0	4.43	36,525	Tb927.10.14360 / Tb427.10.14360	U2A′ (U2-40K) [U2]	698	30	3.60E-66	43.7	475	8	13.8	879	867	1,746
**24**	487	73	3.70E-45	57.1	9.52	36,655	Tb927.3.4880 / Tb427.03.4880	putative nuclear assembly protein	1,096	12	5.50E-106	52.2				380	1,362	1,742
**25**	893	36	1.10E-85	29.0	1.29	110,049	Tb927.1.2570 / Tb427.01.2570	coatomer beta subunit (beta-coP)	835	19	6.10E-80	23.3				697	1,038	1,735
**26**	910	33	2.00E-87	43.1	2.49	59,572	Tb927.11.3240 / Tb427tmp.02.0750	T-complex protein 1, zeta subunit	809	21	2.40E-77	38.1				710	1,005	1,716
**27**	895	35	6.60E-86	35.5	1.95	57,330	Tb927.8.5250 / Tb427.08.5250	coatomer delta subunit	805	22	6.70E-77	33.0				699	1,000	1,699
**28**	864	41	8.60E-83	41.1	1.23	98,250	Tb927.1.120 / Tb427.01.120	retrotransposon hot spot (RHS) protein	815	20	7.20E-78	25.7				675	1,013	1,687
**29**	891	37	1.60E-85	47.8	4.49	41,221	Tb927.10.15180 / Tb427.10.15180	nucleosome assembly protein	779	24	2.40E-74	43.0				696	968	1,664
**30**	798	46	3.50E-76	44.5	2.23	52,847	Tb927.3.1930 / Tb427.03.1930	PRP17 [PRP19 complex]	798	23	3.50E-76	30.8				623	992	1,615
**31**	847	42	4.20E-81	36.7	1.06	97,811	H25N7.12	retrotransposon hot spot protein, RHS4	762	27	1.40E-72	24.8				661	947	1,608
**32**	1,034	26	8.90E-100	33.9	1.57	61,316	Tb927.11.11790 / Tb427tmp.01.3650	R3H domain-containing protein, putative	643	38	9.80E-61	25.8	35	20	3.8	807	799	1,606
**33**	953	30	1.10E-91	44.5	1.77	60,845	Tb927.9.5880 / Tb427tmp.160.4290	SKIP [PRP19 complex]	685	31	7.20E-65	35.6				744	851	1,595
**34**	915	32	7.10E-88	37.7	1.25	71,474	Tb927.6.3740 / Tb427.06.3740	heat shock 70 kDa protein, mitochondrial precursor	685	32	6.40E-65	29.4				714	851	1,565
**35**	918	31	3.30E-88	35.4	1.66	97,586	Tb927.11.11900 / Tb427tmp.01.3740	coatomer gamma subunit	680	34	2.30E-64	28.6				717	845	1,562
**36**	898	34	2.90E-86	61.1	2.56	12,784	Tb927.6.4340 / Tb427.06.4340	SSm2-1 (Sm15K) [U2]	669	35	2.50E-63	61.1	663	7	39.3	701	831	1,532
**37**	883	39	1.00E-84	42.3	3.17	34,615	Tb927.9.15100 / Tb427tmp.244.2730	60S ribosomal protein L5	666	36	5.20E-63	34.3				689	828	1,517
**38**	1,043	25	1.00E-100	35.8	1.93	38,392	Tb927.11.2060 / Tb427tmp.46.0001	60S acidic ribosomal subunit protein	557	48	4.00E-52	27.5				814	692	1,506
**39**	722	51	1.30E-68	73.7	19.45	23,139	Tb927.7.6380 / Tb427.07.6380	SSm4 [U4]	722	28	1.30E-68	64.7	30	21	10.2	564	897	1,461
**40**	844	43	8.40E-81	41.7	2.05	63,028	Tb927.9.9290 / Tb427tmp.211.0930	PABP1	626	41	5.60E-59	35.6				659	778	1,437
**41**	1,050	24	2.30E-101	49.6	5.56	28,405	Tb927.9.15360 / Tb427tmp.244.2630	40S ribosomal protein S6	496	59	4.90E-46	42.6				820	616	1,436
**42**	682	54	1.30E-64	33.7	1.61	53,646	Tb927.4.1310 / Tb427.04.1310	zink finger domain family member, putative	682	33	1.30E-64	31.6				532	847	1,380
**43**	977	29	3.90E-94	32.9	1.21	94,333	Tb927.10.4560 / Tb427.10.4560	elongation factor 2	484	60	7.40E-45	19.3				763	601	1,364
**44**	1,002	27	1.30E-96	49.1	5.56	28,460	Tb927.10.190 / Tb427.10.190	40S ribosomal protein S6	458	64	3.50E-42	37.9				782	569	1,351
**45**	819	45	2.30E-78	61.4	6.08	29,404	Tb11.v5.0501	40S ribosomal protein S3A	572	47	1.20E-53	49.0				639	711	1,350
**46**	750	49	2.20E-71	38.8	3.72	34,557	Tb927.9.13990 / Tb427tmp.211.4540	DRBD2 (RNA-binding protein)	601	43	1.70E-56	35.3				586	747	1,332
**47**	829	44	2.50E-79	43.0	2.90	45,487	Tb927.11.8770 / Tb427tmp.12.0011	eukaryotic initiation factor 4a	535	51	6.10E-50	36.0				647	665	1,312
**48**	644	56	9.10E-61	59.6	23.36	14,027	Tb927.3.1780 / Tb427.03.1780	LSm8 [U6]	644	37	9.10E-61	59.6	50	16	20.9	503	800	1,303
**49**	628	58	3.00E-59	46.8	1.93	34,594	Tb927.11.14900 / Tb427tmp.01.6530	coatomer epsilon subunit	628	40	3.00E-59	39.5				490	780	1,271
**50**	683	53	9.50E-65	53.9	1.94	45,934	Tb927.5.3020 / Tb427.05.3020	SLU7	576	46	4.70E-54	46.9				533	716	1,249
**51**	634	57	8.90E-60	46.0	1.70	58,404	Tb927.10.1060 / Tb427.10.1060	T-complex protein 1, delta subunit, putative	584	45	7.40E-55	32.8				495	726	1,221
**52**	597	63	4.50E-56	85.3	22.88	12,766	Tb927.11.16760 / Tb427tmp.02.2030	ALBA2	597	44	4.50E-56	71.5				466	742	1,208
**53**	709	52	2.70E-67	62.3	7.96	22,424	Tb927.9.5750 / Tb427tmp.160.4250	tryparedoxin peroxidase (TRYP1)	500	57	1.80E-46	55.5				554	621	1,175
**54**	788	47	3.50E-75	46.5	5.31	13,250	Tb927.8.5180 / Tb427.08.5180	LSm2 [U6]	436	71	4.80E-40	46.5	121	13	13.6	615	542	1,157
**55**	602	61	1.30E-56	25.9	1.72	41,174	Tb927.4.3540 / Tb427.04.3540	unknown function, conserved	531	53	1.50E-49	25.9	263	10	17.3	470	660	1,130
**56**	725	50	6.70E-69	100.0	11.74	11,090	Tb927.9.5690 / Tb427tmp.160.4200	60S acidic ribosomal protein	449	67	2.40E-41	100.0				566	558	1,124
**57**	601	62	1.60E-56	39.1	1.48	54,653	Tb927.11.16760 / Tb427tmp.01.8510	T-complex protein 1, alpha subunit, putative	524	55	7.40E-49	31.2				469	651	1,120
**58**	767	48	3.70E-73	32.3	1.27	45,361	Tb927.9.4680 / Tb427tmp.160.3270	Eukaryotic initiation factor 4A-1	409	75	2.60E-37	24.0				599	508	1,107
**59**	545	67	6.00E-51	53.6	4.18	22,377	Tb927.10.11950 / Tb427.10.11950	AD002, CWC15	545	49	4.00E-45	49.6				425	677	1,103
**60**	539	68	2.70E-50	61.2	5.45	21,972	Tb927.10.1100 / Tb427.10.1100	60S ribosomal protein L9	535	50	6.30E-50	58.7				421	665	1,086
**61**	597	64	4.10E-56	35.6	1.14	48,743	Tb927.10.10170 / Tb427.10.10170	PRL1 / PRP46 [PRP19 complex]	497	58	4.10E-46	33.8				466	618	1,084
**62**	650	55	2.20E-61	57.8	5.19	24,735	Tb927.9.8070 / Tb427tmp.211.0110	60S ribosomal protein L10	451	65	1.50E-41	40.5				507	560	1,068
**63**	623	59	1.00E-58	81.2	98.31	12,324	Tb927.2.4540 / Tb427.02.4540	SmB	462	63	1.20E-42	73.2	66	15	26.6	486	574	1,060
**64**	882	40	1.40E-84	43.1	2.60	41,855	Tb927.3.5050 / Tb427.03.5050	60S ribosomal protein L4	280	94	2.20E-24	29.2				689	348	1,036
**65**	568	65	2.90E-53	77.6	12.53	12,481	Tb927.2.5850 / Tb427.02.5850	SmD2	473	61	1.10E-43	77.6				443	588	1,031
**66**	505	70	6.40E-47	37.6	1.09	78,663	Tb927.11.14960 / Tb427tmp.01.6600	pumillo/PUF RNA binding protein 7	505	56	6.40E-47	22.9				394	627	1,022
**67**	555	66	7.10E-52	71.8	11.13	9,649	Tb927.6.2700 / Tb427.06.2700	SmE	442	70	1.30E-40	71.8				433	549	982
**68**	609	60	2.70E-57	45.9	11.92	17,510	Tb927.10.5330 / Tb427.10.5330	40S ribosomal protein S18	380	79	1.10E-35	44.6				475	472	948
**69**	469	74	2.90E-43	43.5	4.32	24,508	Tb927.11.14150 / Tb427tmp.01.5740	SPF27 [PRP19 complex]	462	62	2.90E-43	39.0				366	574	940
**70**	450	77	2.30E-41	45.3	3.20	28,641	Tb927.10.14600 / Tb427.10.14600	40S ribosomal protein S2	450	66	2.30E-41	36.9				351	559	910
**71**	449	78	2.40E-41	44.1	1.90	42,689	Tb927.10.14790 / Tb427.10.14790	metallo-peptidase, Clan MG, Family M24	449	68	2.40E-41	34.6				351	558	908
**72**	447	79	4.10E-41	36.5	1.19	68,785	Tb05.5K5.70	nucleolar RNA helicase II, putative	447	69	4.10E-41	27.2				349	555	904
**73**	434	81	8.40E-40	60.5	34.86	14,732	Tb927.10.4950/Tb427.10.4950	SSm2-2 (Sm16.5K) [U2]	434	72	8.40E-40	60.5	251	11	16.0	339	539	878
**74**	465	75	5.80E-43	49.6	3.40	27,713	Tb927.7.1730 / Tb427.07.1730	60S ribosomal protein L7	412	74	1.40E-37	46.9				363	512	875
**75**	428	82	3.10E-39	28.3	1.26	71,204	Tb927.10.14550 / Tb427.10.14550	ATP-dependent RNA helicase (putative DDX3 homolog)	425	73	5.90E-39	25.3				334	528	862
**76**	517	69	3.60E-48	20.4	1.15	37,804	Tb927.8.3530 / Tb427.08.3530	glycerol-3-phosphate dehydrogenase [NAD]	346	83	5.00E-31	19.5				404	430	834
**77**	404	84	7.60E-37	38.4	2.38	30,401	Tb927.9.6070 / Tb427tmp.160.4450	40S ribosomal protein S3	402	76	1.20E-36	35.5				315	500	815
**78**	438	80	2.90E-40	43.3	9.56	15,512	Tb927.6.4980 / Tb427.06.4980	40S ribosomal protein S14, putative	379	80	2.70E-34	41.9				342	471	813
**79**	400	85	2.10E-36	69.1	4.54	21,505	Tb927.5.990 / Tb427.05.990	unknown function, conserved	400	77	2.10E-36	60.9				312	497	809

**80**	393	86	1.10E-35	35.4	1.60	47,584	Tb927.9.5730 / Tb427tmp.160.4240	nucleosome assembly protein-like protein	393	78	1.10E-35	28.7				307	488	795
**81**	420	83	2.20E-38	55.4	4.57	23,840	Tb927.9.3920 / Tb427tmp.160.2490	ribosomal protein S7	348	82	3.00E-31	42.5				328	432	760
**82**	377	87	3.70E-34	34.2	1.96	41,895	Tb927.9.8850 / Tb427tmp.211.0620	actin A	368	81	2.90E-33	30.5				294	457	752
**83**	489	72	2.80E-45	59.2	2.31	13,571	Tb927.9.5150 / Tb427tmp.160.3670	ribosomal protein S6	294	91	7.60E-26	42.2				382	365	747
**84**	491	71	1.50E-45	51.9	2.22	21,009	Tb927.11.14130 / Tb427tmp.01.5720	ribosomal protein L18	287	93	4.10E-25	42.5				383	357	740
**85**	463	76	1.10E-42	34.2	1.78	19,967	Tb927.5.2910 / Tb427.05.2910	unknown function, conserved	277	95	4.00E-24	22.6				361	344	706
**86**	341	93	1.60E-30	61.4	3.22	14,147	Tb927.11.13960 / Tb427tmp.01.5535	LSm4 [U6]	330	84	1.90E-29	37.3				266	410	676
**87**	327	98	4.20E-29	75.4	3.84	10,207	Tb927.5.4030 / Tb427.05.4030	LSm7 [U6]	327	85	4.20E-29	52.8	41	18	25.0	255	406	662
**88**	326	99	5.50E-29	62.5	7.77	14,962	Tb927.9.11380 / Tb427tmp.211.2630	60S ribosomal protein L23	326	86	5.50E-29	57.4				255	405	660
**89**	358	91	3.00E-32	43.3	2.39	20,087	Tb927.10.560 / Tb427.10.560	40S ribosomal protein S11	287	92	4.10E-25	31.2				279	357	636
**90**	312	102	1.30E-27	47.7	9.78	13,618	Tb927.3.3480 / Tb427.03.3480	U2-B'' [U2]	306	87	5.00E-27	47.7				244	380	624
**91**	306	103	5.00E-27	43.9	1.54	30,866	Tb927.3.3490 / Tb427.03.3490	TDP1, high mobility group protein	306	88	5.00E-27	24.2				239	380	619
**92**	351	92	1.60E-31	49.4	1.24	20,280	Tb927.9.11740 / Tb427tmp.211.2850	PPIL3 (peptidyl-prolyl cis-trans isomerase-like 3 protein)	268	99	3.30E-23	34.2				274	333	607
**93**	300	104	2.00E-26	88.2	3.22	8,356	Tb927.9.10250 / Tb427tmp.211.1695	SmF	295	89	6.10E-26	54.0				234	367	601
**94**	295	105	7.20E-26	44.2	1.13	21,654	Tb927.8.2090 / Tb427.08.2090	PPIL1 [PRP19 complex]	295	90	7.20E-26	34.5				230	367	597
**95**	330	97	2.20E-29	63.9	5.01	20,487	Tb927.9.3480 / Tb427.09.3480	Cwc21 [U5]	265	100	6.30E-23	56.4				258	329	587
**96**	324	100	7.80E-29	43.3	2.39	20,047	Tb927.1.3180 / Tb427.01.3180	40S ribosomal protein S11	253	104	1.00E-21	31.3				253	314	567
**97**	283	106	1.10E-24	42.7	1.44	27,592	Tb927.11.10790 / Tb427tmp.01.2560	40S ribosomal protein SA	273	97	9.40E-24	35.1				221	339	560
**98**	332	96	1.40E-29	38.1	1.54	30,860	Tb927.8.1330 / Tb427.08.1330	60S ribosomal protein L7a	241	105	1.50E-20	31.7				259	299	559
**99**	275	109	7.00E-24	38.2	2.56	19,285	Tb927.10.5360 / Tb427.10.5360	40S ribosomal protein S10	273	96	9.20E-24	33.7				215	339	554
**100**	269	111	2.50E-23	21.6	1.65	33,710	Tb927.10.15410 / Tb427.10.15410	glycosomal malate dehydrogenase	269	98	2.50E-23	18.6				210	334	544
**101**	371	88	1.50E-33	53.7	5.82	16,987	Tb927.7.1040 / Tb427.07.1040	40S ribosomal protein S16	204	110	9.00E-17	45.3				290	253	543
**102**	281	107	1.80E-24	33.5	1.87	39,047	Tb927.6.4280 / Tb427.06.4280	glyceraldehyde 3-phosphate dehydrogenase	260	102	2.10E-22	33.3				219	323	542
**103**	338	94	2.90E-30	45.1	1.83	11,714	Tb927.7.3120 / Tb427.07.3120	SmD1	219	108	2.40E-18	45.1				264	272	536
**104**	366	89	4.80E-33	42.7	4.81	16,222	Tb927.11.11820 / Tb427tmp.01.3675	40S ribosomal protein S17	198	113	2.90E-16	37.3				286	246	532
**105**	258	112	3.60E-22	18.1	1.04	40,677	Tb927.10.13720 / Tb427.10.13720	RBP29, RNA-binding protein, putative	258	103	3.60E-22	18.1				201	321	522
**106**	271	110	1.70E-23	20.9	3.98	15,257	Tb927.10.9800 / Tb427.10.9800	60S ribosomal protein L22	233	106	9.80E-20	20.2				212	290	501
**107**	338	95	3.30E-30	47.6	1.48	18,013	Tb927.4.1100 / Tb427.04.1100	ribosomal protein L21E (60S)	187	119	3.80E-15	33.6				264	232	496
**108**	313	101	1.10E-27	36.6	1.63	16,857	Tb927.11.16130 / Tb427tmp.01.7800	nucleoside diphosphate kinase	197	114	4.50E-16	36.6				244	245	489
**109**	364	90	8.10E-33	35.1	1.24	25,412	Tb927.3.3310 / Tb427.03.3310	60S ribosomal protein L13	137	132	4.10E-10	24.7				284	170	454
**110**	222	115	1.20E-18	24.6	2.30	20,568	Tb927.10.4270 / Tb427.10.4270	coatomer zeta subunit	222	107	1.20E-18	20.4				173	276	449
**111**	209	116	2.40E-17	25.1	1.68	25,040	Tb927.8.6150 / Tb427.08.6150	40S ribosomal protein S8	209	109	2.40E-17	21.1				163	260	423
**112**	234	113	8.40E-20	88.8	5.07	8,917	Tb927.11.14310 / Tb427tmp.01.5915	SmG	193	115	1.10E-15	74.0	36	19	17.1	183	240	422
**113**	208	117	3.00E-17	33.8	1.15	21,469	Tb927.11.11230 / Tb427tmp.01.3020	60S ribosomal protein L14	203	111	9.60E-17	18.9				162	252	415
**114**	201	118	1.60E-16	32.8	1.12	28,482	Tb927.8.2000 / Tb427.08.2000	cyclophilin (PPIL)	201	112	1.60E-16	26.4				157	250	407
**115**	227	114	3.90E-19	33.0	1.04	34,689	Tb927.11.11360 / Tb427tmp.01.3170	receptor for activated C kinase 1	176	122	5.10E-14	28.9				177	219	396
**116**	192	119	1.10E-15	27.6	1.10	39,062	Tb927.11.3830 / Tb427tmp.02.1320	unknown function, conserved	192	116	1.10E-15	25.1				150	239	388
**117**	191	120	1.50E-15	33.0	1.69	20,651	Tb927.9.13650 / Tb427tmp.211.4460	ADP-ribosylation factor	191	117	1.50E-15	26.1				149	237	386
**118**	189	121	2.80E-15	28.1	2.54	16,061	Tb927.10.8430 / Tb427.10.8430	40S ribosomal protein S12	189	118	2.80E-15	21.2				148	235	382
**119**	186	122	4.90E-15	53.7	2.04	22,179	Tb927.8.1110 / Tb427.08.1110	40S ribosomal protein S9	186	120	4.90E-15	41.8				145	231	376
**120**	184	124	8.60E-15	20.6	1.30	34,706	Tb927.10.11230 / Tb427.10.11230	C-terminal homology with human NF-kappa-B-activating proteir	184	121	8.60E-15	20.6				144	229	372
**121**	174	125	7.50E-14	39.9	1.50	22,369	Tb927.9.7590 / Tb427tmp.160.5580	60S ribosomal protein L11	174	123	7.50E-14	19.2				136	216	352
**122**	173	127	1.10E-13	24.9	1.63	21,184	Tb927.10.11390 / Tb427.10.11390	60S ribosomal protein L6	173	124	1.10E-13	21.3				135	215	350
**123**	185	123	6.30E-15	23.4	1.13	21,664	Tb927.9.12200 / Tb427tmp.211.3270	60S ribosomal subunit protein L31	161	125	1.60E-12	18.3				144	200	344
**124**	160	129	2.20E-12	36.5	2.15	21,385	Tb927.11.6300 / Tb427tmp.02.4170	40S ribosomal protein S5	160	126	2.20E-12	30.9				125	199	324
**125**	157	132	3.70E-12	37.1	1.27	14,851	Tb927.11.8890 / Tb427tmp.01.0625	RPC19, subunit of RNA pol I and III	157	127	3.70E-12	37.1				123	195	318
**126**	156	133	5.50E-12	24.9	1.23	25,628	Tb927.10.10010 / Tb427.10.10010	60S acidic ribosomal protein	156	128	5.50E-12	17.6				122	194	316
**127**	157	131	4.20E-12	38.8	1.70	16,454	Tb927.8.6180 / Tb427.08.6180	60S ribosomal protein L26	133	133	9.90E-10	26.8				123	165	288
**128**	140	134	1.90E-10	46.8	1.91	11,253	Tb927.2.2670 / Tb427.02.2670	histone H4	140	130	1.90E-10	35.1				109	174	283
**129**	138	135	3.50E-10	24.8	3.05	11,534	Tb927.10.3370 / Tb427.10.3370	60S acidic ribosomal protein P2	138	131	3.50E-10	24.8				108	171	279
**130**	131	140	1.60E-09	33.7	2.19	14,037	Tb927.11.4460 / Tb427tmp.02.2040	ALBA1	131	134	1.60E-09	28.2				102	163	265
**131**	158	130	3.30E-12	43.2	1.06	16,830	Tb927.11.6200 / Tb427tmp.02.4050	60S ribosomal protein L28	114	137	8.30E-08	22.2				123	142	265
**132**	137	136	3.80E-10	45.9	1.85	15,572	Tb927.11.15880 / Tb427tmp.01.7535	60S ribosomal protein L27	118	136	3.50E-08	25.4				107	147	254
**133**	135	138	6.60E-10	45.4	4.17	12,367	Tb927.10.1590 / Tb427.10.1590	ribosomal protein L36	94	139	0.0000076	42.7				105	117	222
**134**	104	141	7.70E-07	61.3	2.64	12,559	Tb927.3.1370 / Tb427.03.1370	40S ribosomal protein S25	104	138	7.70E-07	31.5				81	129	210
**135**	134	139	8.80E-10	38.3	1.61	16,965	Tb927.4.2180 / Tb427.04.2180	60S ribosomal protein L35a	83	140	0.00011	11.7				105	103	208
**136**	82	143	0.00012	56.6	1.22	10,116	Tb927.7.7380 / Tb427.07.7380	LSm3 [U6]	82	141	0.00012	21.8				64	102	166

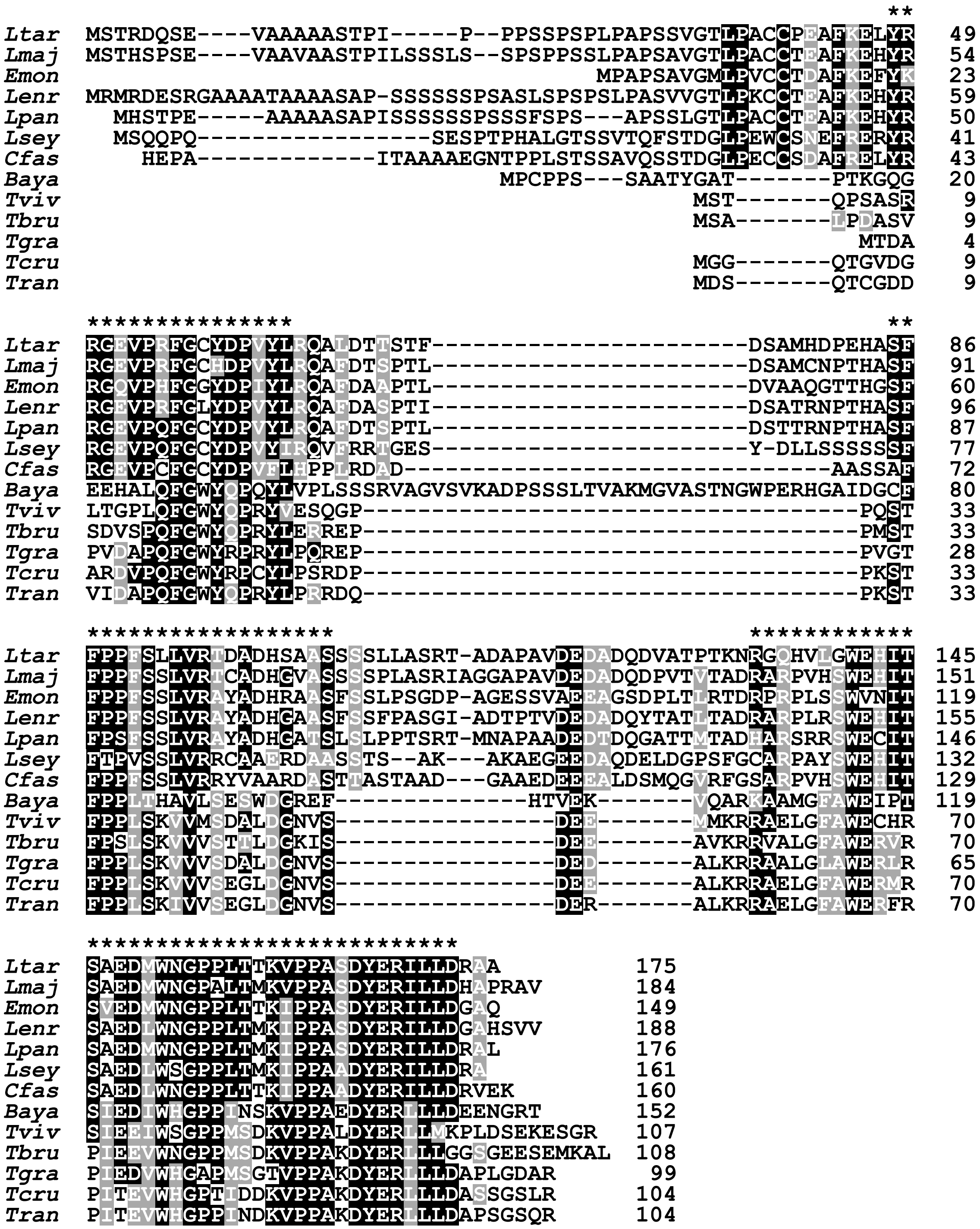

Fig. S1. PRC5 is conserved among trypanosomatids. PRC5 amino acid sequences were aligned using the Clustal Omega server of the European Bioinformatics Institute at default parameters [1]. Positions with more than 50% identity or similarity are highlighted in black and gray, respectively. Dashes indicate sequence gaps and asterisks mark conserved domains. Sequences were obtained from the TriTrypDB data base at www.TriTrypDB.org [2] and are from *Leishmania tarentolae* (*Ltar*, LtaP33.2230), *Leishmania major (Lmaj*, LMJLV39_330030600*),* *Endotrypanum monterogeii* (*Emon*, EMOLV88_330025800), *Leishmania enrietti* (*Lenr*, LENLEM3045_330028800), *Leishmania panamensis* (*Lpan*, LPAL13_330028700), *Leptomonas seymouri* (*Lsey*, Lsey_0022_0070), *Crithidia fasciulata* (*Cfas*, reading frame on chromosome 23, nucleotide positions CfaC1_23:491307-491789, located by the TBLASTN algorithm), *Blechomonas ayalai (Baya,* Baya_003_0830), *Trypanosoma vivax* (*Tviv*, TvY486_1103020), *Trypanosoma brucei* (*Tbru*, accession number Tb927.11.2960), *Trypanosoma grayi* (*Tgra*, Tgr.1410.1000), *Trypanosoma cruzi (Tcru*, TcCLB.510431.330), and *Trypanosoma rangeli* (*Tran*, TRSC58_01412).

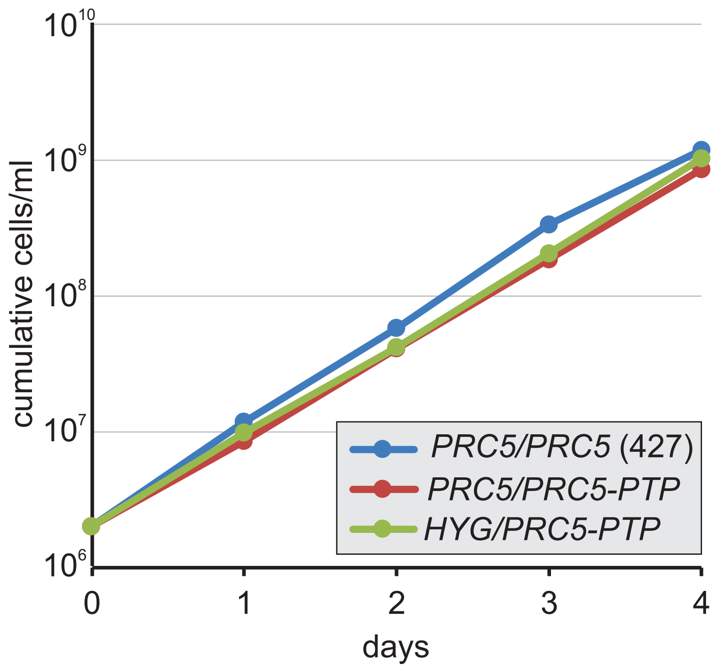

Figure S2. C-terminally PTP-tagged PRC5 is functional. Growth curves of wild -type procyclic Lister strain 427 trypanosomes with unaltered *PRC5* locus (*PRC5/PRC5*), of the same cells after targeted integration of plasmid PRC5-PTP-NEO which fused the PTP coding sequence to the 3′ end of the coding region in one allele (*PRC5/PRC5-PTP*), and of cells in which a second transfection replaced the remaining wild-type allele with the hygromycin resistance mar ker (*HYG/PRC5-PTP*). Cells were counted using a hemocytometer and diluted to 2x 10^6 cells daily.

```
HsDHX8     KLSKQKKQQRLEPLYNRYEEPNAWRISRAFRRR    1220
TbPRP22    KLTKEQRAERLNPILRAWESGNSWRISKQRRRR    1062
```

Figure S3. The C-terminal amino acid sequences of human and trypanosome DHX8/PRP22 are similar. Amino acid sequence alignment of the C-terminal region of human DHX8 (Accession AAH44586) and *T. brucei* PRP22 (Tb927.10.7280). Identical and conserved amino acids are shaded in black and gray, respectively.

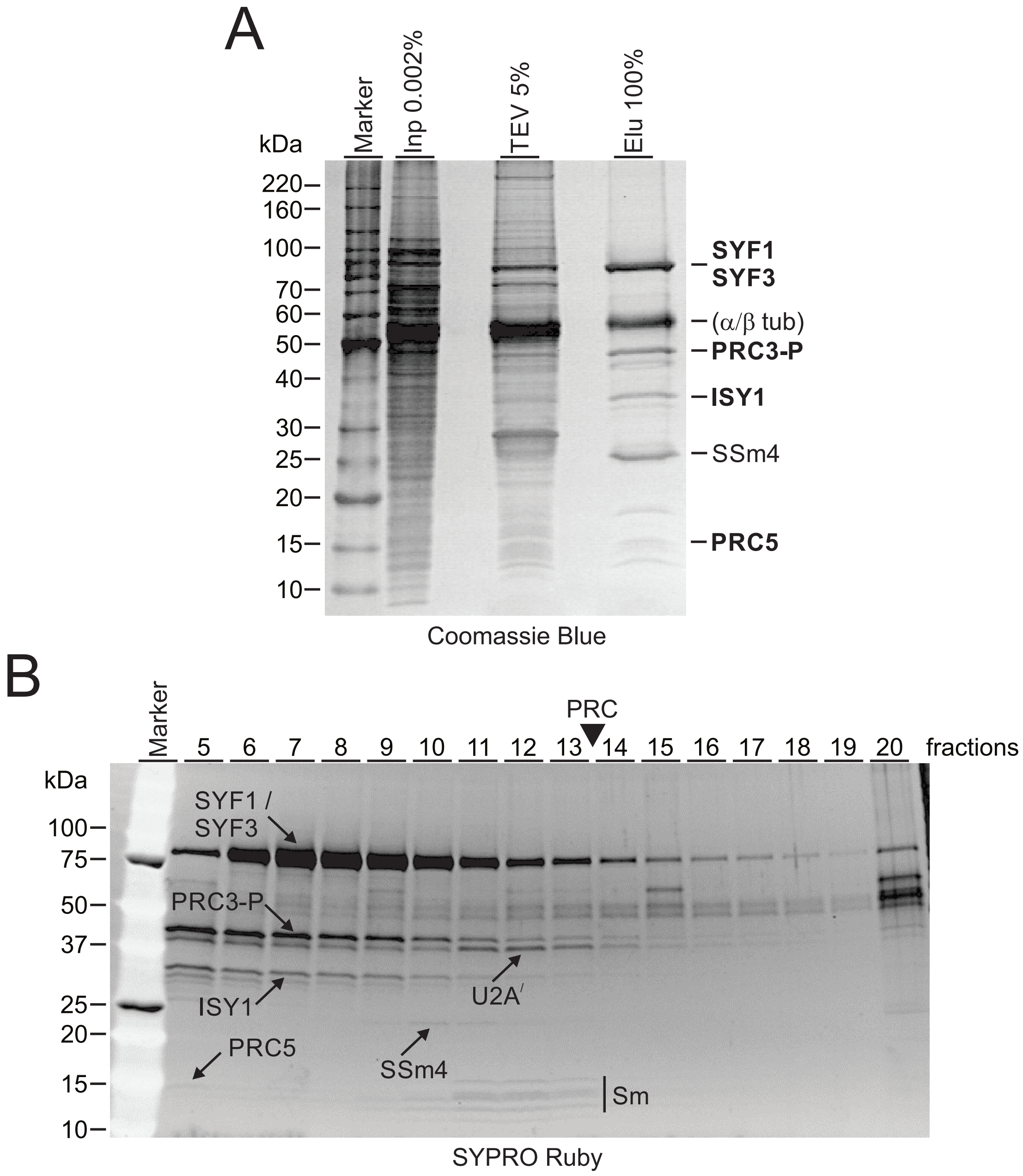

Figure S4. Tandem affinity purification of PRC3-PTP. (**A**) Crude extract (Inp) of procyclic trypanosomes expressing PRC3-PTP, TEV eluate and final eluate (Elu) of a standard PTP tandem affinity purification were separated on a 10–20% SDS polyacrylamide gradient gel and stained with Coomassie blue. The percentages specify relative amounts loaded. (**B**) The final eluate was sedimented through a 10-40% linear sucrose gradient by ultracentrifugation and fractionated from top to bottom into 20 aliquots. Proteins from each fraction were separated by SDS-PAGE and stained with SYPRO Ruby. The sedimentation peak of intact PRC is marked by an arrowhead.

Figure S5 (below). Conservation of the TPR structure in trypanosomatid SYF1 and SYF3 homologs. (**A**) SYF1 amino acid sequences from human (*Hs*; accession NP_064581), *S. cerevisiae* (*Sc*; QHB07883), *T. brucei* (*Tb*; Tb927.5.1340) and *L. major* (Lm; LmjF.23.1550) were aligned using the Clustal Omega server of the European Bioinformatics Institute at default parameters (1). The 15 TPRs, as specified in the human system (2), are numbered and marked by vertical lines. Key consensus residues are specified above each repeat. Positions with more than 50% identity or similarity are highlighted in black and gray, respectively, whereas red highlighting indicates conservation of consensus residues. Dashes indicate sequence gaps. Protein secondary predication was carried out according to the GOR method (3) using the NPS@ server (4) with c, e, and h marking coil, extended or beta strand, and helix positions, respectively. Helix positions within TPRs are marked by dark red lettering. (**B**) Corresponding alignment with SYF3 amino acid sequences from human isoform D (*Hs*; accession NP_001265557), rat (*Rn*; NP_446249), *S. cerevisiae* (*Sc*; NP_013218), *T. brucei* (*Tb*; Tb927.10.9660) and *L. major* (Lm; LmjF.36.4280). The 16 TPRs, as specified in rat (5), are numbered and marked by vertical lines.

A

```
                                                      1
                                                      |..W..Y.........G..
Hs              MVVMARLSRPERPDLVFEEEDLPYEEEIMRN-QFSVKCWLRYIEFKQ--GAPKP---   51
Sc           MSAYIAMKGV-ITNVDENIRNDEDVAFEYEIQKT-PQNILTWKRYIEYWKEEGRTDK---   55
Tb                  MGT-----------TSTDTLELEVLRN-PSNVRGWLQFIRSILCSDYPNQVSK   41
Lm                 MDGD-----------PRVSALQWEVAKEASTSPKTWAELARTTAAATAADPVER   43

Hs helix        ccccceecccccccccceeccccchhhhhhhhh-ceeeecceeeecccc--ccccc
Tb helix            ccc-----------cccchhhhhhccc-ccccccceeeeeeeeeccccccccc

                             2
               .....Y..A....P.||..W..Y.......G...............Y..A....P.|
Hs           --RLNQLYERALKLLPCSYKLWYRYLKARRAQVKHRCVTDPAYEDVNNCHERAFVFMHKM  109
Sc           --QIRWLYERFCSQFVTDTSIWEDYIRWESTKEV--VETSRIFWLFQRCLKS---CVRDC  108
Tb           ANAVNVAYERALRANGYSYKLWMGYISYRRENTRELTSPNEWFRSLRDIYDRAVEKLPMM  101
Lm           ANAVTLVYERALRAFASSYKLWMAYISYRQRETSRMCGPNEWFQAVREVYERALTELPKM  103

Hs helix     --chhhhhhhhhhhhcccccchhhhhhhhhhhhhceeecccccccccccccceeeeeeecc...
Tb helix     cchhhhhhhhhhhhcccceeeceeeeeecccccceeeeccccchhhhchhhhhhhhhhcccc

             3                                      4
             |..W..Y.......   G........Y..A....P.|  |..W..Y.......G......
Hs           PRLWLDYCQFLMDQ---GRVTHTRRTFDRALRAMPITQHSRIWPLYLRFLRSHPLPETAV  166
Sc           DRICLSYLELAIEQ---YDLAMIRHALASSLMKMEREMHRKVWDPVIKFVEEKVLPLTQL  165
Tb           PLLWTSFIEFAMDGSVAPRITLTRHVITRALEALPFTQHHRIWRLAKLWVSRPHVPMPTA  161
Lm           PMLWVSYMEFVVAS-EVPRVTMTRHILARALAALPATQHHHLWKIAKRWCAMPIVPSATV  162

Hs helix     eeeeeeeceeeecc---cceechhhhhhhhhhhchhhhcccceeeeeecccccccchhh
Tb helix     ccccceeeeeecccccccccceeeehhhhhhhhhcchhhhhhhhhhhhhhhccccccccccc

             ..Y..A...     .P.|
Hs           RGYRRFLKL----SPESAEE-------------Y------------------------IE  185
Sc           DSTQEDEEE----STDEAELINVLLVKGFTKGGFISEEI----SENGSRGDIWSSHILER  217
Tb           TYIWRLYLLYDPSTENQRNYFHMLWEKGNA-SDFLVECAAFLLRDSTSHGG------L--  212
Lm           RAVWWLYLSFQRSLHAKREYFQVLVQKGDF-NGFLQECVRLGLPDKSNKGAVEERDLL--  219

Hs helix     hhhhhhhhc----ccchhhh-------------h------------------------hh
Tb helix     eeeeeeeeccccccchhhhhhhhhhcccc-cchhhhhhhhhcccccccc------e--

Hs           YLKSSDRLDEAAQRLATVV----------NDERFVSKAGK-SNYQLWHELC-DLISQNPD  233
Sc           YLKVAPQQK-RNESLATLALTRDNITIKSVYEKYLPQDENSGKYLPSSELPFELNFNYLA  276
Tb           ---------------LRDIA----------FWE-------TVRTALETKGLCFGGDISQVE  241
Lm           ---------------LSDVS----------FWE-------TVQTALQGKGWRFTGDIGRLR  248

Hs helix     hhhchhhhhhhhhhhhhhh----------cchhhhcccc-ccchhhhhhc-cccccccc
Tb helix                    ecchh----------hhh-------hhhhhhhcccccccccchh
```

```
                                    5
                                    |..W..Y.......G........Y..A....P.|
Hs        KVQSLNVD----AIIRGGLTRFTDQLGKLWCSLADYYIRSGHFEKARDVYEEAIRTVMTV  289
Sc        SLEKLGLDNQYEEFMRQMNGIYPDKWLFLIISLAKYYISRGRLDSCGDLLKKSLQQTLRY  336
Tb        K-------------IVQMAADYCASPAEFRLSYAVFLANQGELSMARETLWAILNDVDNP  288
Lm        E-------------LVALGKLRCASPVELSMAFAVFLYGQGHMREGRQELRQLLDEAPEA  295
Hs helix  ccccccee----eeeeccccccccccccccccccceeecccchhhhhhhhhhhhhhhhcc
Tb helix  h-------------hhhhhhcccccchhhhhhhhhhhhhhchhhhhhhhhhhhhcccccc

Hs        RDFTQVFDSYAQFEESMIAAKM---------ETASELGREEEDDVDLELRLARFEQLISR  340
Sc        SDFDRIYNFYLLFEQECSQFILG-----KLKENDSKFFNQKDWTEKLQAHMATFESLINL  391
Tb        AVFCRAFTAALAFESQIIDSLAMDSSIHALDEVKYQQ-LREKLCGDVPDPLYHLTRLTQQ  347
Lm        QTLISLYHLAVEMEDQLVESFAVDPDLRGLDDAAYNG-VVQHLFGD-GDPLRHLERLARE  353
Hs helix  cchhhhhchhhhhhhhhhhhhh---------hhhhhhcccccchhhhhhhhhhhhhhhhh
Tb helix  chhhhhhhhhhhhhhhhhhhhhhhhhhhhhhhhhhhh-hhccccccccccchhhhhhhhc

                          6
                          |..W..Y.......G       ......Y..A.....P.|
Hs        RPLLLNSVLLRQNPHHVHEWHKRVALHQGRP-------REIINTYTEAVQTVDPFKA-T-  391
Sc        YDIYLNDVALRQDSNLVETWMKRVSLQKSA--------AEKCNVYSEAILKIDPRKVGTP  443
Tb        HPMLLNQLQLRADRHCTALWLKRIEILKEMECNGVATSSDVIALYRQAITQCTSGMPNVE  407
Lm        FPLLLNQAQLRNSPHNAALWLKRVELLQEDVYAGRSTFDDLVALYRQAIQQCTAGMSRVD  413
Hs helix  chhhhhhhhccccccchhhhhhhhhhhcccc-------ceeecccccccccccccccc-c-
Tb helix  chhhhhhhhhhhhhhhhhhhhhhhhhhhhcccccccccchhhhhhhhheeeeecccccch

             7                                      8
             |..W..Y.......G........Y..A. ...P.|    |..W..Y.......G...
Hs        GKPHTLWVAFAKFYEDNGQLDDARVILEKAT-KVNFKQVDDLASVWCQCGELELRHENYD  450
Sc        GSFGRLWCSYGDLYWRSNAISTARELWTQSL-KVPYPYIEDLEEIYLNWADRELDKEGVE  502
Tb        AATAQLFESYACYLWENNLRTDAVTVADEGAWFVKFSSTTS--NVLLMGLVVEFSQL-T-  463
Lm        GAAAQLFSSCAQTLIRGGKLDSAIAVLSEGTWCVPFTSAAL--NVQLLGLLVEVQLMSFA  471
Hs helix  cccceeeeeeeeeeeccccchhhhhhhhhhh-hhhhhceeceeeeeeeccccccchhhhh
Tb helix  hhhhhhhhheeeeeecccccccceeeeecccccceeeeeccc--ceeeeceeeeeccc-c-

                                                    9
          .....Y..   A...P.|                        |..W..Y.....
Hs        EALRLLRK---ATALPARRAE----YFDG--SEPVQNRVYKSLKVWSMLADLEE------  495
Sc        RAFSILED---ALHVPTNPEILLEKYKNGHRKIPAQTVLFNSLRIWSKYIDYLEAYCPKD  559
Tb        DPARTLDKLVSRLVKATNVSNSIRS--KGLARQVVVKNLARDPRAWVLAVDVAFHRLLLK  521
Lm        SPLSVRDALAERLTSGARSGASKRSRNGLLQSSAALPRVAHHPHAWIMWCDVCAAY----  527
Hs helix  hhhhhhhh---hhhchhhhhh----cccc--ccccchhhhhhhhhhhhhhhhhh------
Tb helix  cchhhhhhhhhhhhhcccccchhhh--cccchhhhhhccccccchhhhhhhhhhhhhhhh

                                 10
             ..G........Y..A....P.||..W..Y.......G.    .......Y..A....
Hs        ---SLGTFQSTKAVYDRILDLRIATPQIVINYAMFLEEHKY----FEESFKAYERGISLF  548
Sc        ANSSDKIFNKTKMAYNTVIDLRLITPAMAENFALFLQNHYE----VMESFQVYEKTIPLF  615
Tb        NTGESG--GRSNEELKNLISLFCNSSGYTAEGACYVACRLWQSGDVSAAFQEFERALVAF  579
Lm        -----D--SANAAAWHRLVEALASSPAFSAEAACYVAHKAYVCRQESIAVMMLERAVAAT  580
Hs helix  ---hhchhhhhhhhhhhhhhhhhcccchhhhhhhhhhhhhhh----hhhhhhhhhhhhhhh
Tb helix  cccccc--ccchhhhhhheeeecccccceecceeeeeeeeeccccchhhhhhhhhhhhhh

                 11                                           12
          ..P  .||..W..Y....... .G..........Y..A....     P. |  |..W...
Hs        KWP--NVSDIWSTYLTKFIAR-YGGRKLERARDLFEQALDGC-----PP-KYAKTLYLLY  599
Sc        PPE--IQYELWIEYLEVATSHQLSSLSPEHIRFLFEKALKNLC----SNGIDCKTIFIAY  669
Tb        AAAPLAVLHILQQYLSCLCVSFGTRLPLHRFREFSKLGLDVAQFTMRSSPVSTMEFLLNC  639
Lm        RQQPTAQLFVLEQYLSFLCVRDGRQVPLHQFREVFSLVQQVTPVAIRTAPSAVIDLYVSC  640
Hs helix  hcc--ccccchhhhhhhhhhhh-ccchhhhhhhhhhhhccccc-----cc-ccchhhhhh
Tb helix  hhchhhhhhhhhhceeeeeeccccccchhhhhhhhhccceeeeeeeecccceeeeeeeecc

                                                 13
          ....... G........Y..A      .....P.|    |..W..Y........G.....
Hs        AQLEEEW-GLARHAMAVYERA------TRAVEPAQQ---YDMFNIYIKRAAEIYGVTHTR  649
Sc        SVFEERISGLISKSIEILRRGAVIGTVSVSTHLESR---LQLWRMCISKAESTLGPSVTR  726
Tb        VTLESRL-GFSGSAVQIARECLHLAL---KHQAEYDSLLFGVLDAVLEVTFRLHGSQALR  695
Lm        AYMEAAI-GLYGTAVRMMQEISLVATAALRGRDEHLRVLQAILEHSITFTEQRRGYNSVR  699
Hs helix  hhhhhhh-hhhhhhhhhhhhh------hhhhcchhh---hchhhhhhhhhhhhccccccc
Tb helix  eeeeccc-ccccchhhhhhhhhhhhh---hhhhhhhhhhhhhhhhhhhhhhhhcccchhhh
```

```
                                     14                                         15
              ..Y..A...P.      |  |..W..Y.......G........Y..A.....P.|    |.
Hs            GIYQKAIEVLSD-----EHAREMCLRFADMECKLGEIDRARAIYSFCSQICDPRT--GA   702
Sc            ELYQECIQILPN-----SKAVEFVIKFSDFESSIGETIRAREILAYGAKLLPPSRN--TE   779
Tb            ---HYCAELLERQKLTPQLIQRLALWWAAVERRTGNADRAHTVMEACCKSQDPSSSHGAV   752
Lm            ---AYCGALVQRLQ-HALLLQRVALHWAAIEKRSGNTAQAHLILDACGDSQDPSTEHGEV   755
Hs helix      chhhhhhhhhhh-----hhhhhhhhhhhchhhhhccccccchhhhhhccccccccee--cc
Tb helix      ---hhhhhhhhhhcccchhhhhhhhhhhhhhcccccceeeeeeeeeccccccccceee

              .W..Y.......G........Y..A....P.|
Hs            FWQTWKDFEVRHGNEDTIKEMLRIRRSVQATYNTQVNFMASQMLKVSGSATGTVSDLAPG   762
Sc            LWDSFEIFELKHGDKETYKDMLKMKKVLESNMLIDSASVS--------HEEGNINFVAAA   831
Tb            FWSMWESICNT---VKQFEGVHRRKQQAALKYSSGNNSADAAVDGVDGNKNNNVG-----   804
Lm            YWRLWESLCTN---VEEFEKVARRRQQASIRFNKRQSAKGAE×                   794
Hs helix      cccccccceeeccccchhhhhhhhhhhhhhccccceeeeeeeeeeeeecccccceeeecccc
Tb helix      eeeeeecccce---ecccccchhhhhhhhhhhhhcccccccceeeeeccccccccccc-----

Hs            QSGMDDMKLLEQRAEQLAAEAERDQPLRAQSKILFVRSDASREELAELAQQVNPEEIQLG   822
Sc            TSHAPNSHTLTQST----------------------------------SSYSINPDEIELD   858
Tb            ASGNEAIEVVPVPALT×------------------------------------------   820
Hs helix      ccchhhhhhhhhhhhh…
Tb helix      cccccceeeeccceec×

Hs            EDEDEDEMDLEPNEVRLEQQSVPAAVFGSLKED×   855
Sc            I×                                 859
```

B

```
                                        1                                         2
                                        |..W........E.LG..E.A......AL.L.P.      ||.
Hs            MAASTAAGKQRIP------KVAKVKNKAPAEVQITAEQLLREAKERELELLLP----PPPQ   50
Rn            MAASTAAGKQRIP------KVAKVKNKAPAEVQITAEQLLREAKERELELLLP----PPPQ   50
Sc                                  MDTLEPTAVDTHVSAEQILRDVYKKGQK-AR----GSTN   34
Tb            MKRNRNHNDQMGPAAAALRRRSVFRDARASSTQLTDAQLIAGALEMQRSIGPS--NTSTT   58
Lm            MKRSRQDNRRLGPAAAAMRRRDVYRDGRQAEEQVSIQKLFRDAVAMQKFHSPNQSGAGAR   60
Hs helix      cccccccccccch------hhhhhhcccchhhhhhhhhhhhhhhhhhhhhhccc----cccc
Tb helix      cccccccccccchhhhhhhhhhhhhhhhccccchhhhhhhhhhhhhhhhhhcccccc--cccee

                                          3
              .W.....E.LG..E.A......AL.L.P.||..W........E.LG..E.A......AL.L
Hs            QKITDEEELNDYKLRKRKTFEDNIRKNRTVISNWIKYAQWEESLKEIQRARSIYERALDV   110
Rn            QKITDEEELNDYKLRKRKTFEDNIRKNRTVISNWIKYAQWEESLKEIQRARSIYERALDV   110
Sc            IDILDLEELREYQRRKRTEYEGYLKRNRLDMGQWIRYAQFEIEQHDMRRARSIFERALLV   94
Tb            VVINSPEELALYRQKTRAELEERVKRGYTFLGNWVKYARWEAQQKDYERMRSILERAVKF   118
Lm            VMIHSREELALYRQKRRAELEESVRRGFKAIGNWIRYARWEAQQRDFDRMRAVMERAVPV   120
Hs helix      cccchhhhhhhhhhhhhhhchhhhhccceeeeeechhhhhhhhhhhhhhhhhhhhhhhhhccc
Tb helix      eeeccchhhhhhhhhhhhhhhhhhhhhceeeeechhhhhhhhhhhhhhhhhhhhhhhhhhhhh

                  4                                 5
              .P.||..W........E.LG..E.A......AL.L.P.||..W........E.LG..E.A..
Hs            DYRNITLWLKYAEMEMKNRQVNHARNIWDRAITTLPRVNQFWYKYTYMEEMLGNVAGARQ   170
Rn            DYRNITLWLKYAEMEMKNRQVNHARNIWDRAITTLPRVNQFWYKYTYMEEMLGNVAGARQ   170
Sc            DSSFIPLWIRYIDAELKVKCINHARNLMNRAISTLPRVDKLWYKYLIVEESLNNVEIVRS   154
Tb            HGANPVLWRDYAELEEEGGFINHARAVWERGVTALPSATDLWLKYIVLEQAAGQDGKVRD   178
Lm            HGENANLWRDYAELEESNGFAEHARQVWSRGVTALPSSVDLWVKYLAMEQAAGQDQRVRD   180
Hs helix      cccchhhhhhhhhhhhhhhhhhhccccchhhhhhcccccccceeeeeeeehhhhhhhhchhh
Tb helix      ccccchhhhhhhhhhhcccchhhhhhhhhhhcccccccccchhhhhhhhhhhhcccccceee

                      6                                 7
              ....AL.L..P||..W........E.LG..E.A......AL.L.P||..W........E. L
Hs            VFERWMEW-QPEEQAWHSYINFELRYKEVDRARTIYERFVLVHPDVKNWIKYARFEEK-H   228
Rn            VFERWMEW-QPEEQAWHSYINFELRYKEVERARTIYERFVLVHPAVKNWIKYARFEEK-H   228
Sc            LYTKWCSL-EPGVNAWNSFVDFEIRQKNWNGVREIYSKYVMAHPQMQTWLKWVRFENR-H   212
Tb            LFNRWVSGPTPPACAWELFALFEAQQRRAENCRDILRRYVEAHGTVECWIFYGSAELNVL   238
Lm            IFHRWLAGDAVPPCAYALAALYEAQQRRQAGCRDILRRCVERFNTPASWLLYGATEQQVF   240
Hs helix      hhhhhhhh-cccccchhhhhhhhhhhhhhhhhhhhhhhhhhhhheeeecccccchhhhhhhhhh-h
Tb helix      eeeeccccccccccchhhhhhhhhhhhhhhhhhhhhhhhhhhhhhhhheeecccceeeeeeeeccccchhhh
```

```
                                       8
         G..E.A......AL.L.P.|          |..W.......E.LG..E.A......AL.
Hs       AYFAHARKVYERAVEFFGDEH---------MDEHLYVAFAKFEENQKEFERVRVIYKYALD  280
Rn       AYFAHARKVYERAVEFFGDEH---------MDEHLYVAFAKFEENQKEFERVRVIYKYALD  280
Sc       GNTEFTRSVYSLAIDTVANLQNLQIWSDMEVAKLVNSFAHWEAAQQEYERSSALYQIAIE  272
Tb       KNADRAAMVYACAMESLPNEY---INGE-KDCRIPLAWADALAAALKFDEARELYHRLLK  294
Lm       GDHERAVRVLETAMHALPDED---LWGL-EECRVPLALAEAHVAAGNAVQARNVFHNALE  296
Hs helix hhhhhhhhhhhhhhhhccccc---------chhhhhhhhhhhhhhhhhhhhhhhhhhhhhh
Tb helix hhhchhhhhhhhhhhcccccce---eccc-cccchhhhhhhhhhhhhhhhhhhhhhhhhhhhh

                   9                                      10
         L.P.|     |..W.......E.LG.........E.A......AL.L.P.||..W.....
Hs       RISKQD--AQELFKNYTIFEKKFGDRRGIEDIIVSKRRFQYEEEVKANPHNYDAWFDYLR  338
Rn       RISKQE--AQELFKNYTIFEKKFGDRRGIEDIIVSKRRFQYEEEVKANPHNYDAWFDYLR  338
Sc       KWPSN----QLLKAGLLDFEKQFGDINSIEETISYKRKMEYETILSNNAYDYDTWWLYLD  328
Tb       ECTS-VKALDAIFTAYKRFERLYGSAENHETMTSVIARAMYQHRIAHNPRDFDAYVAQFL  353
Lm       HVADHPVLLEKVLASYSRFERLYGDGAHSEQVARLLTIQLYEERLRTNTSDFDAYLTLYS  356
Hs helix hhhhhh--hhhhhhhhhhhhhhccccccccchhhhhhhhhhhhhhhcccccccchhhhhhh
Tb helix ccch-hhhhhhhhhhhhhhhhhhccccccchhhhhhhhhhhhhhhhhhhccccchhhhhhhhhh

         ..E.LG..E.A.                                    .....AL.L.P.
Hs       LVESDAEAEAVR------------------------------------EVYERAIANVPP  362
Rn       LVESDAEADTVR------------------------------------EVYERAIANVPP  362
Sc       LISESFPKQ-IM------------------------------------QTFEKAIVDSRP  351
Tb       LFRDATQGDNASVCAGHA-VNVGSKGGAW---EEALQCLTAA----------AGIRIDGE  399
Lm       LYRDQERESRLRASGSSNKMPEPVVGGNAAPASAALATSTAAGASPSRQVLERALQ-IPA  415
Hs helix hhhhhhhhhhhh------------------------------------hhhhhhhccccc
Tb helix hhhcccccccceeeecce-eeeccccccch---hhhhhhhhhh----------hcccccch

                        11                                 12
           |            |..W.......E.LG...E.A......A-L.L.P.|    |..W....
Hs       --IQEKRHWKRYIYLWINYALYEELEAKDPERTRQVYQAS-LELIPHKKFTFAKMWILYA  419
Rn       --IQEKRHWKRYIYLWVNYALYEELEAKDPERTRQVYQAS-LELIPHKKFTFAKMWLLYA  419
Sc       KELSKNVQWKRYIYLWMRYICYVELELENSLLEEELFQRLIDDIIPHKHFTFSKIWLMYA  411
Tb       KDAVL---AQHHAVVVMAYARLVEERGDVAAARSALAK--CIRHFPFKTACCPRLWIEAA  454
Lm       RDPYG---AQQRAVLIMEYARACEADGELDAARRTLAQ--EIKNFPFDVALCPRLWVEAA  470
Hs helix --chhhhcceeeeeeehhhhhhhhhhhhccchhhhhhhhhh-hhhchhhhhhhhhhhhhhh
Tb helix hhhhh---hhhhhhhhhhhhhhhhhhhhhhhhhhhhhhhhh--hheeeceeeeccccchhhhh

                                         13
        ...E.LG..E.A......AL.L.P.||.W.......E.LG.
Hs       QFEIRQKNLSLARRALGTSIGKCPKNKLFKVYIELELQLRE-------------------  460
Rn       QFEIRQKNLPFARRALGTSIGKCPKNKLFKGYIELELQLRE-------------------  460
Sc       KFLIRHDDVPKARKILGKAIGLCPKAKTFKGYIELEVKLKE-------------------  452
Tb       ALEERHGEFVQAQKLLAASLNISPCPEVFDAALRLESNACA-------------------  495
Lm       AMEERHGATAQARRLLRAGSQVTKDACLFEEAIQFEERQRDSVMEAMHAAAHETTPPSAR  530
Hs helix hhhhhhhhhhhhhhhccccccccccccchhhhhhhhhhhh-------------------
Tb helix hhhhhhhhhhhhhhhhhhhhccccccccchhhhhhhhhhhhhh-------------------

                                     14
                  .E.A......AL.L.P.||..W.......E.LG..E.A......AL.L.P.
Hs       ---------FDRCRKLYEKFLEFGPENCTSWIKFAELETILGDIDRARAIYELAISQPRL  511
Rn       ---------FDRCRKLYEKFLEFGPENCTSWIKFAELETILGDIERARAIYELAISQPRL  511
Sc       ---------FDRVRKIYEKFIEFQPSDLQIWSQYGELEENLGDWDRVRGIYTIALDENSD  503
Tb       AGEVSAESCVQRSRAIYESAIKQFPQNFALWEGYAKLEEKERQFHRSDALRAACIRSFSA  555
Lm       EVEEACAPYVAELRADYQNAIQAFPFDVQWWLRYAKLEEQQHEILRADALYGACIRTFND  590
Hs helix ---------hhhhhhhhhhhhhhcccccchhhhhhhhhhhhhhhhhhhhhhhhhhhhhcccc
Tb helix ccccchhhhhhhhhhhhhhhhhhccccchhhhhhhhhhhhhhhhhhhhhhhhhhhhhhhhhh

              15
             |   |..W.......E.LG..E.
Hs       ----DMPEVLWKSYIDFEIEQEETER----------------------------------  533
Rn       ----DMPEVLWKSYIDFEIEQEETER----------------------------------  533
Sc       FLTKEAKIVLLQKYITFETESQEFEK----------------------------------  529
Tb       VARS--VVSLSERYEVLEKVDQAWARRLALKRRLLRSARK-------KTCGGLSESAEVE  606
Lm       EAER--VRSFAQRYDLLSHVDAAWAHRIHLHTRRARALQRQASRRKGNAAGAETARAEVA  648
Hs helix ----ccchhhhhhhhhhhhhhhhhh-----------------------------------
Tb helix hhhh--hhhhhhhhhhhhhhhhhhhhhhhhhhhhhhhhhhhhcccc-------ccccccchhhhh
```

```
                                            16
             A......AL.L.     P ||..W.......E.LG.....E
Hs       ------TRNLYRRLLQRT----Q-HVKVWISFAQFELSSGKEGSLT----------------  568
Rn       ------TRNLYRQLLQRT----Q-HVKVWISFAQFELSSGKEGSVA----------------  568
Sc       ------ARKLYRRYLELN----QYSPQSWIEFAMYQTSTPTEQQLLDLAKLQ---------  571
Tb       ELNFSLRQLYQELLDSVWDEYIYEALRWRGKNA-----STVG-VLSPPPLRLPEVLTPAT  660
Lm       ALQEELLHLYPTFLQDVWNAYRHEAVAWFSRYFEAGEGSGSGVLAGAAPKTLPASLTPAT  708
Hs helix -----hhhhhhhhhhhc----c-ceeeeeeeeeeecccccccch---------------
Tb helix hhhhhhhhhhhhhhccchhhhhhhhhhhhccccc-----ceee-ecccccccccccchhh

                             .A              ......A                 L
Hs       ---------------------KC------------RQIYEEA-----------------N  578
Rn       ---------------------KC------------RQIYEEA-----------------N  578
Sc       ---SENVDEDIEFEITDENKLEA------------RKVFEEA-----------------I  599
Tb       ARWAEAVSAVTNFVVS-STTADGRP--EDSSVEWMRTTFRKRLERYRPKLLQEFGGLDNN  717
Lm       ARWSEAVEAVAGYMER-MSAIKAGQGASTGATAALRAILKSMVEAERHAIRRSLGWTEQT  767
Hs helix ---------------------hh------------hhhhhcc-----------------c
Tb helix hhhhhhhhccceeeeee-cccccccc--ccchhhhhhhhhhhhhhhchhhhhhcccccccc

         .L.P.|
Hs       KTMRNCEEKE------ERLMLLESWRSFEEEFGTASDKERVDKLMPEKVKKRRKVQTDDGS  633
Rn       KTMRNCEEKE------ERLMLLESWRSFEDEFGTVSDKERVDKLMPEKVKKRRKVQADDGS  633
Sc       VEFKEKDDKQ------GRLSILEALKDYEETYGTELDQETVKKRFPKVIKKVRL---QNG-  650
Tb       ASFEAIQQSKRWVEFLLSPNGTEWRRFEAAHGTSDTLEAAKECTQPTARRTRLFVKKKA×  776
Lm       TQFDAVQRAREWGELLLSPLLEEWSKFELTHGGSLEAVA-AAMEKPVKRRTRLFKARS×  824
Hs helix hhhhhhhhhh-----hhhhhhhhhhhhhhhhccchhhhhhhhhhhhhhheeeeecccc
Tb helix chhhhhhhhhhheeeeccccccchhhhhhhccccchhhhhhhccccchhhhheeeeeec

Hs      DAGWEEYFDYIFPEDAANQ--PNLKLLAMAKLWKKQQQEKEDAEHHPDEDVDESES×     687
Rn      DAGWEEYYDYIFPEDAANQ--PNLKLLAMAKLWKKQQQEREAAEQDPDKDIDESESSSF×  690
Sc      --VEEEFVDYIFPDDIDDDKPKPSKFLELAKKWKQEQAL×                      687
```

```
                        **********************   ***********************
Tbru  MSATP[16 aa]GSQLLLRKFPLLAKARAYAQET---PDDPRAECNLLCYGSDVYRIYSL  66
Tcon     MS[16 aa]GSDLLLRKLPLLAKAYALAQQH---PKDPQAECNLLCYGPEVYRILST  63
Lmaj  MASAS[14 aa]GSDLLLRKVPLYAQARRYAAVD---PDDPQSEGNTLGYGPSSYPLLAA  64
Cfas      M[14 aa]GSELLLRKVPIYAQARQYATVD---ADDAQSEGNTLGYGVSSYPLLAC  60
Pcon  MPRFS[28 aa]PSQVLLGKLAIRSRLQRALADMADTGGDTPASRRLSFGQEMYPHLFF  81

      ***                         ****************
Tbru  TSA[15 aa]QR[174 aa]TEGTGGDAVRSYLRRVAWIEQQKGRQSLTNSAAETAPHGE 300
Tcon  ARR[12 aa]QR[187 aa]DVEQDGEAVQSYLRRVSWIEQQKKLQLGLGAATGTRHHSE 307
Lmaj  VRS[42 aa]QR[209 aa]EEVEQQQKVLAYLKRVRWIATQWSSTTNQM-AFKDAGASA 359
Cfas  VRT[44 aa]QR[178 aa]EEVDQQQKVLAYLKRVRWIATQWSSASAAS-SLKDA-AAA 325
Pcon  LRE[39 aa]QR[212 aa]QNTATTEQEQTYLRRVRWLERQRQLNTLTS------GASG 362

        *****************************
Tbru  QLWSNMTEAARVQKEIMRMRAQRKQFHRERKKGE                       334
Tcon  QPWMSMTDAMRVQKEMQRMRLQRKRFHRPHKSTEG                      342
Lmaj  PSWGQMTESQRVKAEMLHAQGQRRRFRRPRRGGDDGAKGNGEDADAALENELGSA 414
Cfas  PSWSQLSEAARVKTEMQHTQGQRRRFRRVRRGPADGSANTD-DVDATEE        373
Pcon  VSWMAMRESERVQRTMSRIEGKRKRFRREKQ                          393
```

Figure S6. PRC3 is conserved among trypanosomatids in its 5′- and 3′-terminal regions. PRC3 amino acid sequences from *T. brucei* (*Tbru*, accession number Tb927.2.3400), *T. cruzi (Tcru*, TcCLB.508771.40), *L. major (Lmaj*, LmjF.33.2880), *Crithidia fasciculata* (*Cfas*, CFAC1_210040400), and *Paratrypanosoma confusum* (*Pcon*, PCON_0011290) were aligned using the Clustal Omega server of the European Bioinformatics Institute at default parameters (1). Positions with more than 50% identity or similarity are highlighted in black or gray, respectively. Dashes indicate sequence gaps and asterisks mark conserved domains. In brackets and blue lettering, a region's number of amino acids (aa) are specified that exhibit no conservation among the sequences.

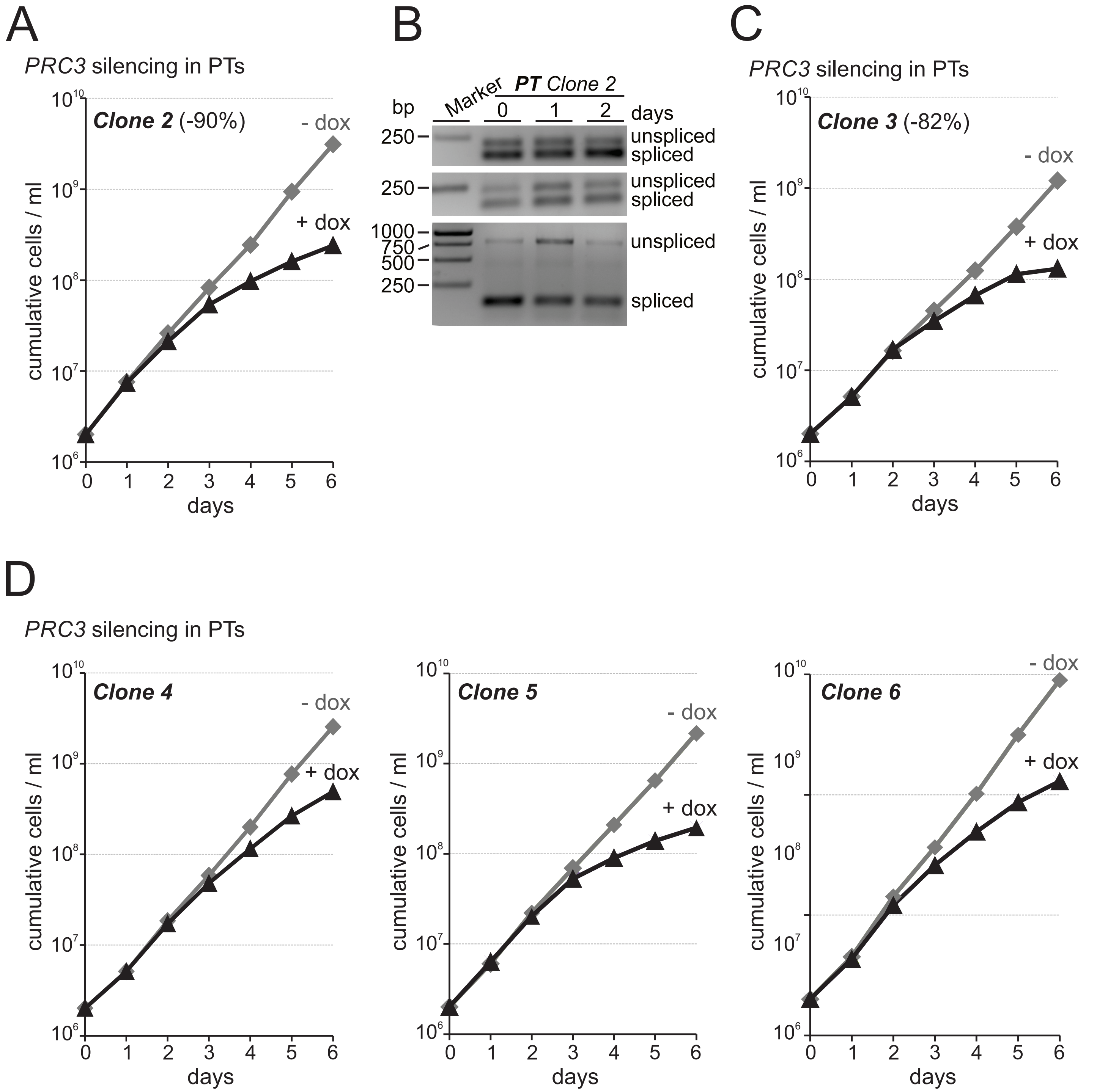

Figure S7. Analysis of clonal procylic trypanosome (PT) lines in which *PRC3* was conditionally silenced. (**A**) Culture growth of clone 2 in which the *PRC3* mRNA level dropped by 90% after 1 day of *PRC3* silencing, i.e. doxycycline (dox) treatment. (**B**) RT-PCR analysis of α-tubulin (top) and *PAP1* (middle) *trans* splicing and *PAP1 cis* splicing (bottom) splicing in RNA preparations of doxycycline-treated clone 2 cells. (**C**) Culture growth of clone 3 without and with doxycycline treatment, the latter of which reduced the *PRC3* mRNA level by 82% after 1 day. (**D**) Culture growth of clones 4-6 for which RNA was not analyzed.

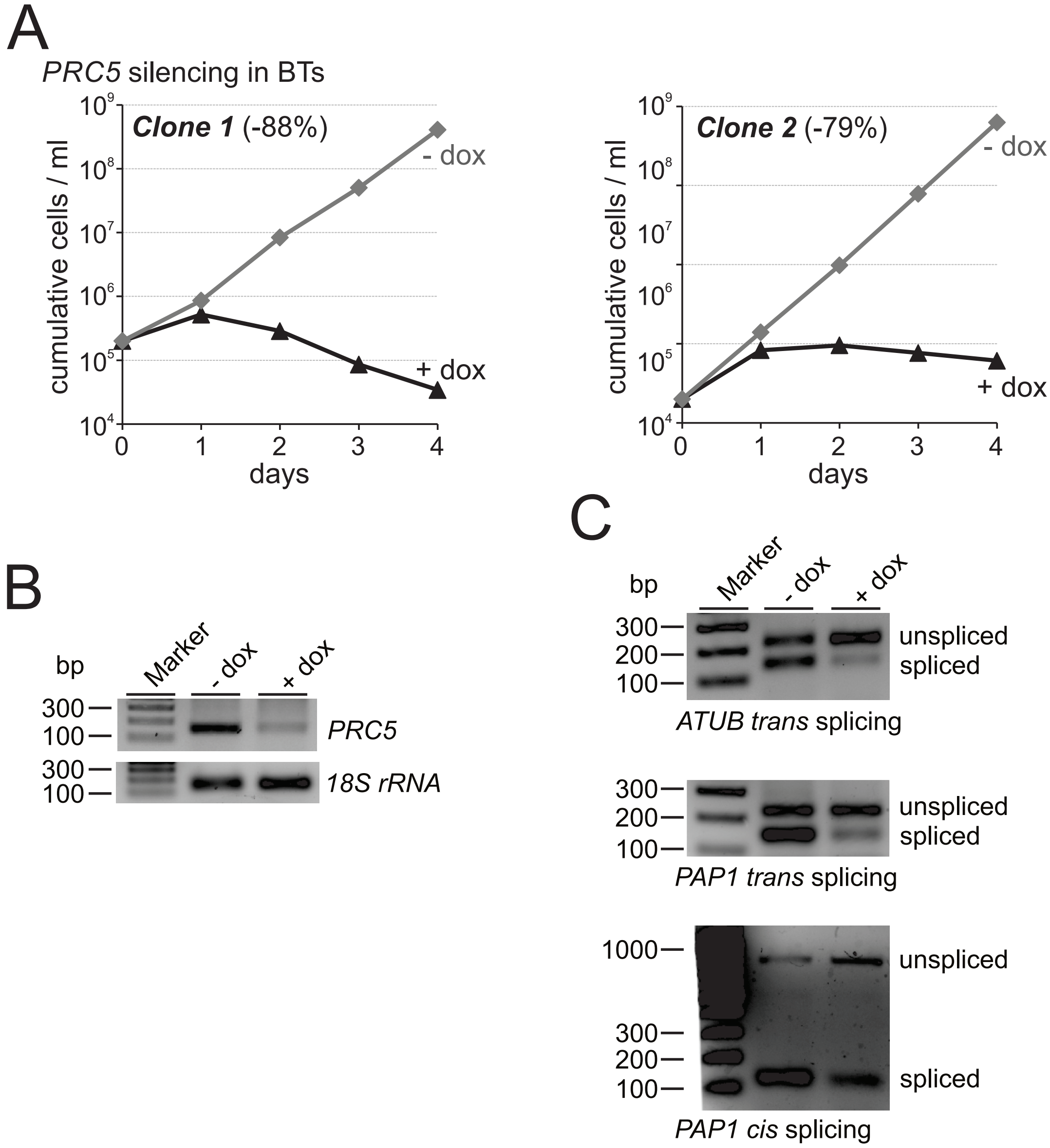

Figure S8. Analysis of clonal bloodstream trypanosome (BT) lines in which *PRC5* was conditionally silenced. (**A**) Culture growth of clones 1 and 2 in which the *PRC5* mRNA level dropped by 89 and 79%, respectively, after 1 day of *PRC5* silencing, i.e. doxycycline (dox) treatment. (**B**) Total RNA was prepared from clone 1 that was either uninduced (- dox) or treated with doxycycline for 1 day (+ dox). RNA was reverse-transcribed with random hexamers and *PRC5* and 18S rRNA sequences amplified by semi-quantitative PCR. (**C**) The same cDNA was analyzed by 3-primer and 2-primer PCR assays that showed α tubulin (*ATUB*) and *PAP1* RNA *trans* splicing and *PAP1 cis* splicing defects.

Supplemental References

1. Sievers F, Wilm A, Dineen D, Gibson TJ, Karplus K, Li W, *et al*. (2011) Fast, scalable generation of high-quality protein multiple sequence alignments using Clustal Omega. Mol Syst Biol 7: 539.
2. Nakatsu, Y, Asahina H, Citterio E, Rademakers S, Vermeulen W, Kamiuchi S, Yeo JP, Khaw MC, Saijo M, Kodo N *et al.* (2000) XAB2, a novel tetratricopeptide repeat protein involved in transcription-coupled DNA repair and transcription. *J Biol Chem*, **275**, 34931-34937.
3. Garnier J, Gibrat JF and Robson B (1996) GOR method for predicting protein secondary structure from amino acid sequence. *Methods Enzymol*, **266**, 540-553.
4. Combet C, Blanchet C, Geourjon C and Deleage G (2000) NPS@: network protein sequence analysis. *Trends Biochem Sci*, **25**, 147-150.
5. Amada N, Tezuka T, Mayeda A, Araki K, Takei N, Todokoro K and Nawa H (2003) A novel rat orthologue and homologue for the *Drosophila* crooked neck gene in neural stem cells and their immediate descendants. *J Biochem*, **133**, 615-623.
