# Supplementary material for: A distinct complex of PRP19-related and trypanosomatid-specific proteins is required for pre-mRNA splicing in trypanosomes
Source: Nucleic Acids Res. 2021 Dec 1;49(22):12929–42. doi: 10.1093/nar/gkab1152 (PMC8682746; doi:10.1093/nar/gkab1152)
Supplement: gkab1152_Supplemental_Files [file gkab1152_supplemental_files.zip › Table S2 data caption.docx]

**Table S2 data.** Summary of complete mass spectrometry data derived from final eluates of two independent PRC5-PTP tandem affinity purifications.
